# Chidamide increases the sensitivity of refractory or relapsed acute myeloid leukemia cells to anthracyclines via regulation of the HDAC3 -AKT-P21-CDK2 signaling pathway

**DOI:** 10.1186/s13046-020-01792-8

**Published:** 2020-12-09

**Authors:** Hao Wang, Yu-chen Liu, Cheng-ying Zhu, Fei Yan, Meng-zhen Wang, Xiao-su Chen, Xiao-kai Wang, Bao-xu Pang, Yong-hui Li, Dai-hong Liu, Chun-ji Gao, Shu-jun Liu, Li-ping Dou

**Affiliations:** 1 Department of Hematology, Chinese People’s Liberation Army (PLA) General Hospital, 28 Fuxing Road, Beijing, 100853 China; 2 State Key Laboratory of Inorganic Synthesis and Preparative Chemistry, International Joint Research Laboratory of Nano-Micro Architecture Chemistry (NMAC), International Research Center for Chemistry-Medicine Joint Innovation, College of Chemistry, Jilin University, 2699 Qianjin Street, Changchun, 130012 China; 3 School of Medicine, Nankai University, 94 Weijin Road, Tianjin, 300071 China; 4 Department of Orthopedics, Xiqing Hospital, 403 Xiqing Road, Yangliuqing, Tianjin, 300000 China; 5 Department of Cell and Chemical Biology, Leiden University Medical Center, Leiden, the Netherlands; 6 The Hormel Institute, University of Minnesota, 801 16th Avenue NE, Austin, MN 55912 USA

**Keywords:** Chidamide, Histone deacetylase, Histone deacetylase inhibitors, HDAC3, Refractory or relapsed acute myeloid leukemia, PI3K-AKT signaling pathways

## Abstract

**Background:**

Induction therapy for acute myeloid leukemia (AML) is an anthracycline-based chemotherapy regimen. However, many patients experience a relapse or exhibit refractory disease (R/R). There is an urgent need for more effective regimens to reverse anthracycline resistance in these patients.

**Methods:**

In this paper, Twenty-seven R/R AML patients with anthracycline resistance consecutively received chidamide in combination with anthracycline-based regimen as salvage therapy at the Chinese PLA General Hospital.

**Results:**

Of the 27 patients who had received one course of salvage therapy, 13 achieved a complete response and 1 achieved a partial response. We found that the HDAC3-AKT-P21-CDK2 signaling pathway was significantly upregulated in anthracycline-resistant AML cells compared to non-resistant cells. AML patients with higher levels of HDAC3 had lower event-free survival (EFS) and overall survival (OS) rates. Moreover, anthracycline-resistant AML cells are susceptible to chidamide, a histone deacetylase inhibitor which can inhibit cell proliferation, increase cell apoptosis and induce cell-cycle arrest in a time- and dose-dependent manner. Chidamide increases the sensitivity of anthracycline-resistant cells to anthracycline drugs, and these effects are associated with the inhibition of the HDAC3-AKT-P21-CDK2 signaling pathway.

**Conclusion:**

Chidamide can increase anthracycline drug sensitivity by inhibiting HDAC3-AKT-P21-CDK2 signaling pathway, thus demonstrating the potential for application.

## Background

Currently, the treatment for acute myeloid leukemia (AML) includes standard chemotherapy with anthracycline and cytarabine [1, 2]. Although treatment outcomes in adult AML patients have improved over the past decade, up to 30% of adult patients fail to achieve complete remission (CR) following 2 cycles of intensive chemotherapy. In addition, a large number of patients experienced relapse despite having achieved complete remission [3–5]. Therefore, clinicians continue to find it challenging to manage these two types of patients [6–9]. Furthermore, as the exact mechanism for drug resistance remains largely unclear, there is an urgent need to discover the mechanisms for drug resistance and develop more effective treatment regimens for patients with refractory/relapsed AML.

As a promising class of anticancer drugs, histone deacetylase (HDAC) inhibitors induce apoptosis and suppress tumor cell growth [10, 11]. Chidamide is a novel oral HDAC inhibitor designed to inhibit the activity of HDAC1, 2, 3 and 10. Chidamide is currently being used in multiple clinical trials as monotherapy or combination therapy for the treatment of various hematological and solid cancers [12–16]. In these trials, chidamide inhibited cell growth in a variety of cancers, such as lung cancer, pancreatic cancer, leukemia, and lymphoma [15–18]. Chidamide has also inhibited the viability of MDS and AML cells [19]. However, the possibility and ways of using chidamide as a treatment option for relapsed/refractory AML following anthracycline therapy remain to be explored. This study shown that chidamide-based regimen improves the overall remision rate of patients with relapsed/refractory AML, as well as prolongs disease-free survival time and overall survival rates. Moreover, this study is the first to find out chidamide increases the sensitivity of anthracycline-resistant cells to anthracycline drugs, and these effects are associated with the inhibition of the HDAC3-AKT-P21-CDK2 signaling pathway, thus demonstrating the potential for application.

## Materials and methods

### Analysis of clinical cases

Our dataset consists of 27 patients with relapsed/refractory AML, who had received anthracycline-based treatment regimen as induction therapy between Jan. 01, 2018 and Jan. 01, 2019 at the Chinese PLA General Hospital (Supplementary Table 1). According to NCCN (The National Comprehensive Cancer Network) clinical practice guideline for AML, relapse following CR is defined as the reappearance of leukemic blasts in the peripheral blood or the finding of more than 5% blasts in the bone marrow, not attributable to any other cause (bone marrow regeneration after consolidation therapy) or extramedullary relapse. Primary refractory or resistant disease is defined by being unable to achieve complete remission after 1 to 2 cycles of intensive induction therapy.

The earliest time at which AML was diagnosed was on Jun. 01, 2016. Patients who relapsed or were resistant to anthracycline were treated with chidamide (30 mg on days 1, 4, 8, and 11) in combination with an anthracycline-based regimen as salvage therapy. The salvage therapy was as follows: ① chidamide, 30 mg, oral, days 1, 4, 8, and 11; DAC, 20 mg/m^2^/d, i.v., days 1–5; Acla, 20 mg/d, i.v., days 1, 3, 5; Ara-C, 100 mg/m^2^, q12h, i.h., days 1–5; G-CSF, 300 μg/d, i.h., day 0 until neutrophil levels return to normal. ② chidamide, 30 mg, days 1, 4, 8, and 11; Acla, 20 mg/d, i.v., days 1, 3, 5; Ara-C, 100 mg/m^2^, q12h, i.v., days 1–5; G-CSF, 300 μg/d, i.h., day 0 until neutrophil levels return to normal. ③ chidamide, 30 mg, days 1, 4, 8, and 11; mitoxantrone, 5 mg/d, i.v., days 1–5; Ara-C, 100 mg/m^2^, i.h., days 1–5; VP-16, 100 mg/d, i.v., days 3–5; sorafenib, 400 mg, oral, q12h. This study was approved by the ethics committee of the Chinese PLA General Hospital. Patients have given written informed consent for the collection of clinical data.

### RNA-sequencing and data analysis

Total RNA was extracted using TRIzol Reagent (Invitrogen, USA) according to the manufacturer’s instructions. RNA-seq was performed with a Genome Analyzer IIx (Illumina, San Diego, CA, USA). Expression levels were measured using the RPKM method, and the distribution of gene expression was analyzed.

### TCGA database analysis of HDAC3 prognosis

AML patient datasets were obtained from The Cancer Genome Atlas database (TCGA) [20], which had integrated clinical and RNA-seq information. Written informed consent was obtained from all patients and approved by the Human Studies Committee at Washington University [20]. Patients in the top quartile of HDAC3 expression were classified as high-HDAC3, and patients in the bottom quartile of HDAC3 expression were classified as low-HDAC3.

### Drugs and cell lines

Chidamide was received at no cost from Shenzhen Chipscreen Biosciences Ltd. (Shenzhen, China). K562, K562/A02 (multi-drug resistant leukemic cells, chronic myelogenous leukemia transferred to acute myelogenous leukemia), HL60 and its parallel anthracycline-resistant cell line, HL60/ADR, were kindly gifted by Tianjin Institute of Hematology. The human acute myeloid leukemia were also from Tianjin Institute of Hematology. THP-1 cells were purchased from ATCC (American type culture collection) and THP-1/ADR were its parallel anthracycline-resistant cell line. HEK293T was obtained from ATCC.

The cells were maintained at 37 °C in a humidified atmosphere containing 5% CO_2_. K562/A02 cells were cultured in RPMI-1640 media, supplemented with 10% fetal bovine serum (FBS), 100 μg/mL penicillin, and 10 μg/mL streptomycin. HEK293T cells were cultured in DMEM with 10% fetal bovine serum, 2 mM L-glutamine and antibiotics. To maintain drug resistance, doxorubicin was added to the media (final concentration of 0.5 μg/mL) for at least 1 week every 2 months. One week prior to experiments, cells were re-cultured without doxorubicin [21].

Peripheral blood mononuclear cells (PBMCs) from 22 patients, who had resistance to anthracyclines-based treatment regimens, were isolated with Ficoll-Hypaque centrifugation, and cultured in RPMI-1640 media supplemented with 10% fetal bovine serum (FBS), 100 μg/mL penicillin, and 10 μg/mL streptomycin. PBMCs were treated with different doses of chidamide as monotherapy or the combination of chidamide + doxorubicin for 24 h and 48 h, respectively. The basic characteristics are shown in Table 1. All patients have given informed consent for the use of their cells in this study.

**Table 1:** Patients characteristics

Number	Diagnosis	Relapse or refractory	sample	Blast	Treatment
1	AML-M2	Relapse	PB	91.2%	MA, DCAG
2	AML-M2	Relapse	PB	87.5%	CAG, IA,
3	AML-M4	Relapse	PB	85.6%	DCAG, MA
4	AML-M5	refractory	PB	98.7%	DA, DCAG

*Abbreviations*: *PB* peripheral blood, *MA* mitoxantrone + cytarabine, *DA* doxorubicin +cytarabine, *DCAG* decitabine + aclacinomycin +cytarabine +granulocyte colony stimulating factor, *CAG* aclacinomycin + cytarabine + granulocyte-colony stimulating factor;

### Cell proliferation assays

Cell viability was measured using CCK-8 assay (Dojindo Molecular Technologies, Inc) according to the manufacturer’s instructions. Cells were treated with different concentrations of chidamide as monotherapy or the combination of chidamide + doxorubicin for 24 and 48 h, respectively. Subsequently, 10 μL of CCK-8 was added to each well and incubated for 2–3 h. OD values were measured with a microplate reader at 450 nm.

### Cell-cycle analysis

Following 24 and 48 h, cells were harvested and washed once or twice with PBS and fixed with ethanol overnight. After fixation, the cells were washed with PBS, treated with 100 g/mL RNase A, and stained with 100 g/mL PI. Cell-cycle data were collected on a flow cytometer with 488 nm laser and analyzed with MoFlo MLS sorter (Dako, FortCollins, CO).

### Apoptosis assays

At 24 and 48 h after drug treatment, the cells were harvested, washed twice with ice-cold PBS, and resuspended in binding buffer containing 10 uL PI and 5 uL Annexin-V-FITC (YEASEN) for 15 min at room temperature in a light-protected chamber. All specimens were analyzed on a FACS Calibur.

### Real-time PCR

Total RNA was extracted by TRIzol (Invitrogen, Carlsbad, CA, USA) and cDNA was synthesized by PrimeScript™ RT reagent Kit (Takara) according to the manufacturer’s instructions. Real-time PCR was then performed using KAPA SYBR FAST q-PCR Master Mix (2x) Kit using the primers specified in Table 2. We used the 2-ΔΔCt formula to examine the relative quantification of the target genes.

**Table 2:** Primers sequences (5′-3′)

Gene	Forward primers sequences	Reverse primers sequences
HDAC1	AACTgCTAAAgTATCACCAgAggg	TggCCTCATAggACTCgTCA
HDAC2	TCCTgAggTggTTTggTggC	ATATCACCgTCgTAgTAgTAgCAgA
HDAC3	ATgCCTTCAACgTAggCgATg	CgAgggTggTACTTgAgCAgC
HDAC10	TCggCAggATTTgACTCAgC	TggACTCTAgggCACTCTgAC
AKT	AAgTCATCgTggCCAAggAC	AggTggAAgAACAgCTCgC
P21	gAgCTgCgCCAgCTgAggTgT	gACATggCgCCTCCTCTgAgTgCC
CDK2	TggACACgCTgCTggATg	AATggCAgAAAgCTAggCCC
GAPDH	CTCTggTAAAgTggATATTgT	ggTggAATCATATTggAACA

### Western blot analysis

After cells were harvested, proteins were separated by SDS polyacrylamide gel electrophoresis (SDS-PAGE) and transferred onto a polyvinylidene difluoride membrane. The blots were blocked with 5% non-fat dry milk at 37 °C for 2 h and incubated with a specific primary antibody and secondary antibody according to the manufacturer’s instructions.

### HDAC activity assay

HDAC activity was detected using a Colorimetric HDAC Activity Assay Kit (BioVision). Each reaction was conducted with 100 μL, which contained 50 μg of proteins extracted from cells. HDAC activity was measured with a microplate reader (SpectramMaxM5) at 405 nm.

### Plasmid and transfection

HDAC3 shRNA plasmid (sc-35,538-SH, Santa Cruz Biotechnology, City, State, Country) was transfected into cells using Superfect reagent (Qiagen) according to the manufacturer’s protocol. Transfected cells with non-specific shRNA plasmid were used as blank control. HA-HDAC3 plasmid (NM_003883-HA) and its corresponding control plasmid were obtain from Genechem. Flag-AKT and its control plasmid were purchase from Hanbio. Plasmids and their respective control were transfected into cells using Lipofectamine™ 3000 reagent (ThermoFisher #L3000008). After transfection, cells were subjected to RT-PCR and Western blot to verify HDAC3 or ATK expression.

### Co-immunoprecipitation

HEK293 cells were transfected with Flag-AKT (wild type) alone or plus HA-HDAC3 for 48 h. And lysates immunoprecipitated with Flag were immunoblotted for Acetylated-lysine, HA, p-AKT and Flag. Co-immunoprecipitation reagents (ThermoFisher, #88804) were obtain from ThermoFisher.

### In vivo study

The xenograft experiments were performed in NOD/SCID immunodeficient mice (aged 6–8 weeks). The mice were maintained in an air-conditioned pathogen-free room under conditions of controlled lighting (12 h light and 12 h dark per day) and fed a standard diet of laboratory rodent food and water. 1*10^7^ cells K562/A02 cells in 200 μL PBS were injected subcutaneously into the lateral flanks of mice. Tumors were observed and measured every other day. The tumor volumes were determined by the formula V = 0.5*Length*width^2^. The tumor-bearing mice were randomized into 4 groups (*n* = 5, Figure S3A). The mice in doxorubicin-treated group were injected doxorubicin at the dose of 5 mg/kg intraperitoneally once a week and intragastrically instillated normal saline at the same time. The mice in chidamide-treated group were intragastrically instillated at the dose of 5 mg/kg chidamide three times a week and injected with PBS. And the mice in group control were treated both with PBS and normal saline as a control. The mice in the combined-therapy treated group received both intraperitoneal injection of doxorubicin and intragastric instillation of chidamide. Mice were sacrificed at the 21st day after inoculation and tumors were harvested for molecular characterizations. All animal experiments complied with the international and institutional rules and all animal protocols were approved by the Experimental Animal Ethics Committee of Chinese PLA General Hospital.

### Statistical analysis

To determine gene expressions, the threshold of q-value was set at 0.05, and the absolute value of log_2_ ratios at KEGG and GO enrichment using the database for Annotation. Modified Fisher’s exact test was used to analyze the significance of GO and KEGG enrichment. Data obtained from triplicated experiments were reported in mean ± SD and analyzed using SPSS18.0 software. An appropriate Mann–Whitney test or Student’s t-test was adopted to perform comparisons. Kaplan–Meier method and log-rank test were used to analyze the relationship between HDAC3 expression levels and OS or EFS. Cox proportional hazard models were performed to test the associations between HDAC3 expression levels, OS and EFS using multivariate analysis. All data were analyzed using the R 3.1.1 software, and *P*-values below 0.05 were considered to be statistically significant. (*P < 0.05, **P < 0.01, ***P < 0.001).

## Results

### Combination of chidamide with anthracycline-based treatment for R/R AML patients

To test the effects of the combination of chidamide and anthracycline in vivo, twenty-seven patients with R/R AML received the combination therapy of chidamide and anthracycline-based treatment regimen between Jan.01, 2018 and Jan. 01, 2019 at the Chinese PLA General Hospital. Patient characteristics are presented in Table 3. Thirteen were female and fourteen were male, and the median age was 51 (range, 12–71) years. Ten (37.04%) patients had relapsed AML and seventeen (62.96%) patients had refractory AML. Pre-treatment levels of median marrow blasts, white blood cells, hemoglobin, and platelets were 27.6 (2.50–97.0)%, 3.95 (1.40–49.13) × 10 /L, 96 (71–120) g/L, and 101 (16–226) × 10^9^ /L, respectively. Among 27 patients, 3 patients had FLT3-ITD mutation, 3 patients had DNMT3A mutation, 1 patient had BCOR mutation, 2 patients had NRAS mutation, and 1 patient had STAG2 mutation. Nine patients had documented poor-risk cytogenetics, as defined by the NCCN guidelines for AML.

**Table 3:** Patients characteristics

Characteristics	N	%
Patient age, median (range)	51 (12–71)	
Patient gender		
Male	14	51.85
Female	13	48.15
Relapse	10	37.04
Refractory	17	62.96
Diagnosis (WHO)		
M1	0	0
M2	4	14.81
M4	8	29.63
M5	12	44.44
M6	2	7.41
unavailable	1	3.71
Hb, ×g/L median (range)	96 (71–120)	
WBC, ×10^9^/L median (range)	3.95 (1.40–49.13)	
Platelets, × 10^9^/L median (range)	101 (16–226)	
BM blasts median (range)	27.6 (2.50–97.0)	
karyotype-risk (*n*=)^a^		
Favorable-risk	7	25.93
Intermediate-risk	1	3.70
Poor-risk	9	33.33
Unavailable	10	37.04
DNMT3A mutational status		
Wild-type DNMT3A	18	66.67
DNMT3Amutation	3	11.11
Missing	6	22.22
TP53 mutational status		
Wild-type TP53	19	70.37
TP53 mutation	2	7.41
Missing	6	22.22
FLT3-ITD mutational status		
Wild-type FLT3-ITD	18	66.67
FLT3-ITD mutation	3	11.11
Missing	6	22.22
BCOR mutational status		
Wild-type BCOR	20	74.08
BCOR mutation	1	3.70
Missing	6	22.22
NRAS mutational status		
Wild-type NRAS	19	70.37
NRAS mutation	2	7.41
Missing	6	22.22
STAG2 mutational status		
Wild-type STAG2	20	74.08
STAG2 mutation	1	3.70
Missing	6	22.22

*Abbreviations*: *BM* bone marrow, *WBC* white blood count, *HB* hemoglobin, *PLT* Platelet

^a^Cytogenetic groups were defined as follows: favourable – t(8;21), inv. (16), irrespective of the presence of other abnormalities; adverse – monosomy5, monosomy 7, del(5q), abnormal 3q, complex (5 or more chromosomal abnormalities); intermediate – all other abnormal karyotypes, normal karyotype

**Fig. 1:**
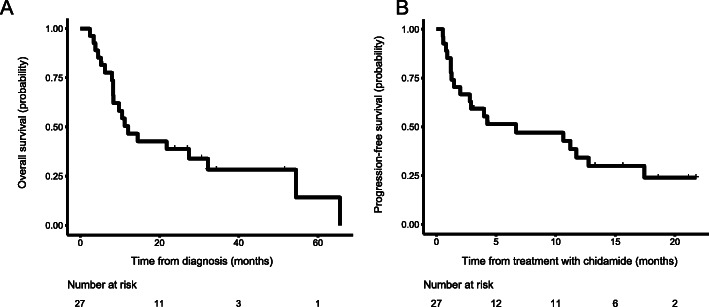
Kaplan-Meier estimates of survival rates of R/R AML (acute myeloid leukemia) patients following combination therapy of chidamide and anthracycline-based regimen. **a** OS. **b** Progression-free survival

Of the 27 patients who had received one course of salvage therapy, 13 achieved a complete response and 1 achieved a partial response. OS rates for 1 and 3 years were 50.44% (95% CI, 34.47–73.81%) and 28.29% (95% CI, 14.68–54.52%), respectively (Fig. 1a). Progression-free survival (PFS) rates for 6 months and 1 year were 51.36% (95% CI, 35.46–74.38%) and 34.24% (95% CI, 19.85–59.06%), respectively (Fig. 1B). Following salvage therapy with a combination of chidamide and anthracycline-based regimen, 26 patients showed grade IV bone marrow suppression, and 1 patient showed grade III bone marrow suppression. The lowest WBC was 0.49 (0.02–1.08) × 10^9^/L and the lowest platelet count was 17 (2–45) × 10^9^/L. The duration of IV suppression was 8 (2–28) days for leukocytes and 8 (2–19) days for platelets. During the treatment, 1 patient reported a new case of pulmonary fungal infection and 2 patients experienced skin infections. Other adverse events include diarrhea, grade I drug-induced liver damage, cholecystitis, and sepsis, with 1 patient reporting each of the events.

### The HDAC3-AKT-P21-CDK2 cell signaling pathway is activated in anthracycline-resistant cells compared to non-resistant cells

HL60, THP-1 and K562 are doxorubicin sensitive cell lines, while HL60/ADR, THP-1/ADR and K562/A02 cells are doxorubicin nonsensitive cell lines. In order to verify the characteristics of drug resistance, we exposed HL60 and HL60/ADR cells to different concentrations of doxorubicin for 24 h, examined the inhibitory activities of doxorubicin on both cell lines by using CCK-8 method. THP-1, K562 and its parallel anthracycline-resistant THP-1/ADR or K562/A02 cells were treated in the same way. As shown in (Supplemental Figure 1A-1B), doxorubin showed different activity in inhibition proliferation of HL60/ADR and HL60 cells, with the IC50 to be 4.818 μg/ml and 0.194 μg/ml, respectively. Compared with HL60 cell, HL60/ADR has 25.4 fold resistance to doxorubin. The IC50 of K562 and K562/A02 were 0.79 μg/ml and 25.462 μg/ml. Compared with K562 cell, K562/A02 has 32.2 fold resistance to doxorubin (Figure S1C-D). Compared with THP-1 cell, THP-1/ADR has 29.4 fold resistance to doxorubin (Figure S1E-F). These results suggested that HL60/ADR, THP-1/ADR and K562/A02 had the characteristics of doxorubin drug resistance.

This study shown that the chidamide-based regimen improves the overall remision rate of patients with relapsed/refractory AML, so we want to further analyze the mechanism of bring such good clinical therapeutic results.

**Fig. 2:**
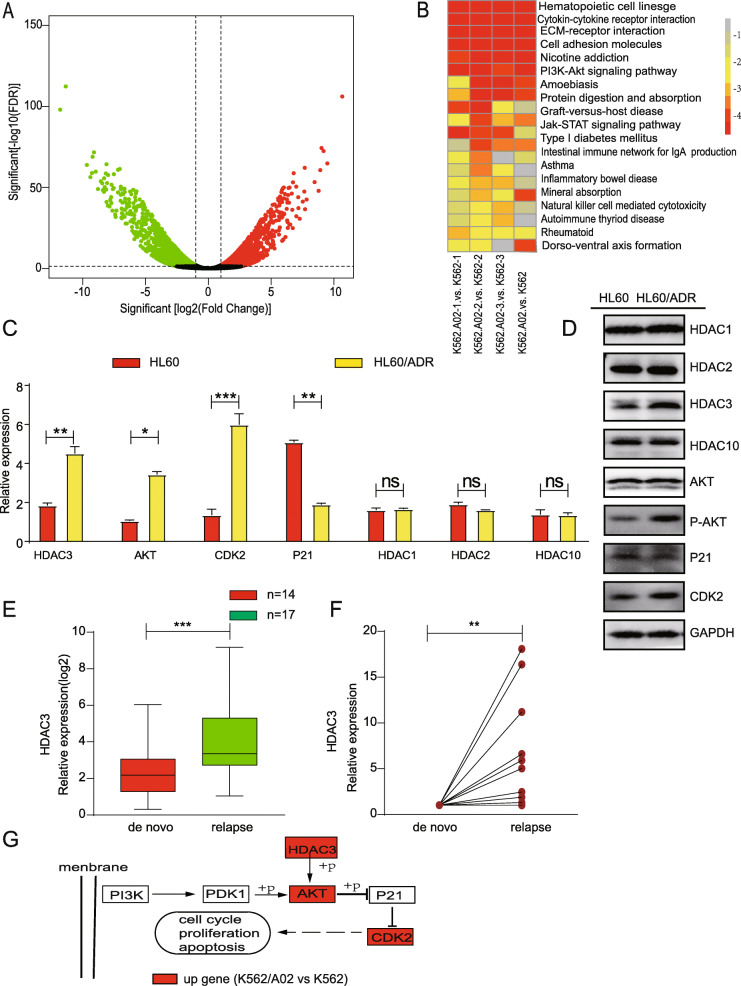
Differential gene and protein expression in K562 and K562/A02. **a** Total RNA isolated from K562 and K562/A02 was subjected to RNA-sequencing. The Volcano plot of differentially expressed gene level was defined by analysis of variance. **b** KEGG pathway analyes of differentially expressed genes. **c** RT-PCR analysis showed differential gene expression in HL60 and HL60/ADR. **d** Western blot analysis shows protein expression of differential gene in HL60 and HL60/ADR. **e** HDAC3 expression of relapsed patients measured with RT-PCR was compared to that of de novo counterparts. **f** The HDAC3 expression of relapsed samples from the same patients measured with RT-PCR was compared to that of its de novo leukemia samples. **g** Compared with K562 cells, the HDAC3-AKT-P21-CDK2 signaling pathway was activated in K562/A02 cells (red represents higher expression). Data represents three independent experiments, and results shown are in the format, mean ± S.D. (NS: *P* > 0.05, *P < 0.05, **P < 0.01, ***P < 0.001)

To study the mechanism of drug resistance in leukemia cells, we performed RNA sequencing (RNA-seq) on K562 cells and K562/A02 (doxorubicin-resistant leukemia) cells. As shown in the Volcano plot (Fig. 2a), RNA-seq identified 368 up-regulated and 171 down-regulated genes in K562/A02 cells. The HDAC3 gene was shown to be one of the highly expressed genes in K562/A02, as compared to that in K562 cells. Drug resistance was closely associated with different cell signaling pathways (with reference to literature), while the PI3K-AKT cell signaling pathway was significantly different between K562 and K562/A02 (Fig. 2b). Within the PI3K-AKT cell signaling pathways, the up-regulated genes were AKT, CDK2, but P21 expression was not significantly different. To further verify the RNA-seq results, we compared the differential gene expression between HL60/ADR and HL60, K562/A02 and K562 cells using quantitative PCR. The expressions of HDAC3, AKT and CDK2 genes were higher in HL60/ADR (Fig. 2c) and in K562/A02 (Figure S2A), while the expression of P21 significantly decreased (Fig. 2c; Figure S2A). These results supported the RNA-seq results. It was previously reported that HDACs can affect the proliferation of malignant tumor cells by altering the expression of oncogenes or tumor suppressor genes [22]. Moreover, aberrant expression of HDACs may protect cells from genotoxic insults [23]. Therefore, we examined whether the HDAC1, HDAC2, and HDAC10 genes were also differentially expressed. However, we did not find significant changes in their expression levels (Fig. 2c, d; Figure S2A, S2B).

To determine whether proteins within the HDAC3-AKT-P21-CDK2 signaling network were differentially regulated, we examined the expression of HDAC3, P-AKT and CDK2 in HL60/ADR and K562/A02 cells. The expression of HDAC3, P-AKT and CDK2 proteins was higher in HL60/ADR (Fig. 2d) and K562/A02 (Fig. S2B), while that of P21 and AKT protein was similar in HL60 and HL60/ADR cells, K562 and K562/A02 cells (Fig. 2d; Figure S2B). Given the close relationship between HDAC3 and AKT [24, 25], our results, which showed that HDAC3 and P-AKT are positively correlated, support the idea that the activation of HDAC3-AKT-P21-CDK2 signal pathways may contribute to anthracycline resistance. This study is the first to find out HDAC3-AKT-P21-CDK2 signal pathways is related to anthracycline resistance .

### High expression levels of HDAC3 are associated with poorer prognoses

The above results suggest that high expression levels of HDAC3 were related to anthracycline resistance. HDAC3 expression of primary leukemia cells from 17 AML patients (de novo samples and relapsed samples) were detected by q-PCR. The expression of HDAC3 in relapsed patients was significantly higher than that in de novo counterparts (Fig. 2e). Interestingly, the expression level of HDAC3 in the relapsed samples from the same patient was higher than that in his de novo samples (Fig. 2f). The qPCR results of the patients showed that the expression of HDAC3 was significantly higher in anthracicycline-resistant cells.

**Fig. 3:**
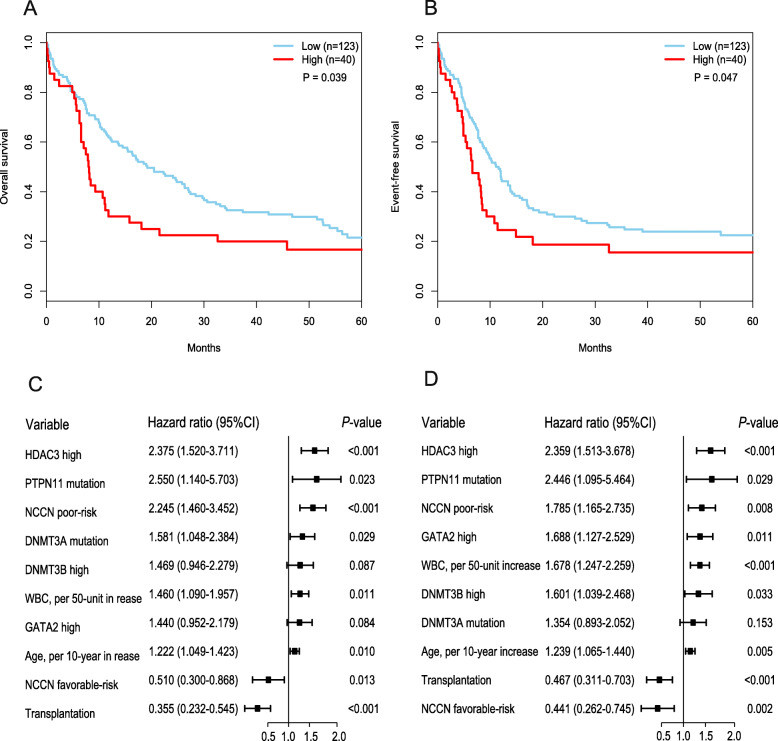
High expression levels of HDAC3 are associated with poorer prognoses**. a** OS and (**b**) EFS in the primary cohort of 163 AML patients. Multivariable analysis of HDAC3 expression associated with OS(**c**) and EFS (**d**)

Then we examined whether high expression levels of HDAC3 are associated with poorer prognoses. We analyzed a cohort of 163 patients with de novo AML from the TCGA dataset. These patients were divided into HDAC3^*Low*^ and HDAC3^*High*^ groups according to their expression levels of HDAC3. This analysis showed that patients with HDAC3^*High*^ had lower median OS (Fig. 3a) and EFS time (Fig. 3b). We also performed multivariate Cox proportional hazard models to analyze the prognosis of HDAC3 expression. In the multivariate analyses, HDAC3^*High*^ expression had an adverse impact on OS (Fig. 3c) and EFS (Fig. 3d).

### Chidamide synergizes with anthracycline drugs which potently inhibited tumor growth and suppressed HDAC3-AKT-P21-CDK2 signaling pathway in vivo

To further validate the combination effect of chidamide with doxorubicin in vivo, we established a xenograft mouse model by subcutaneously inoculating K562/ADR cells into NOD/SCID mice (total = 20). The mice were randomized into 4 groups (*n* = 5). And the mice in the doxorubicin-treated group were injected doxorubicin at the dose of 5 mg/kg intraperitoneally once a week and intragastrically instillated normal saline at the same time. The mice in the chidamide-treated group were intragastrically instillated at the dose of 5 mg/kg chidamide three times a week and injected with PBS at the same time. And the mice in group control were treated both with PBS and normal saline as a control. The mice in the combined-therapy group received both intraperitoneal injection of doxorubicin and intragastric instillation of chidamide (Figure S3A). The volume of tumors in the control group and only doxorubicin treated group was larger than that of tumors in the combination group (Figure S3B). The tumor volumes and the tumor weights at the terminal point were extremely smaller in the combined group than in the control and doxorubicin groups (Figure S3C-E). Moreover, the mRNA expression of HDAC3, AKT, CDK2 decreased and P21 increased in chidamide treated group (Figure S3F). These results indicated that chidamide combined with doxorubicin could significantly inhibit tumor growth.

### Chidamide potently inhibits the viability of anthracycline-resistant AML cells

**Fig. 4:**
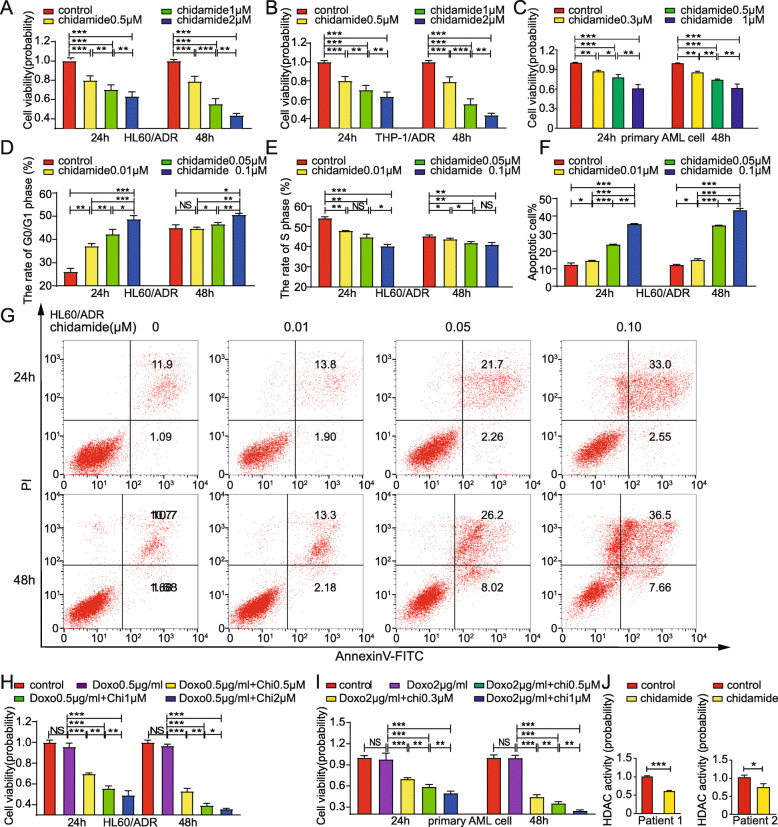
HL60/ADR, THP-1/ADR and patient-derived anthracycline-resistant AML cells are sensitive to either chidamide monotherapy or combination therapy of chidamide + doxorubicin**.** CCK-8 assays were used to assess the abilities of HL60/ADR (**a**), THP-1/ADR (**b**) and patient-derived cells (**c**) to proliferate. HL60/ADR G1 phase (**d**) and proportion of S phase (**e**) in response to incubation with chidamide were measured using flow cytometry. **f-g** are representatives of flow cytometry (Annexin V/PI) for detection of apoptosis. The cell viability of (**h**) HL60/ADR and (**i**) patients-derived primary AML cells treated by combination therapy were measured with CCK-8 assays. HDAC activity of anthracycline-resistant AML cells which were treated with chidamide (**j**). Data represents three independent experiments, results are shown in the format, mean ± S.D. (*P < 0.05, **P < 0.01, ***P < 0.001, NS: *P* > 0.05)

To determine whether chidamide inhibits anthracycline-resistant AML cell growth, cell viability was determined using the CCK-8 assays. HL60/ADR cells, K562/A02 cells and THP-1/ADR cells were treated with 0, 0.5, 1, or 2 μM chidamide for 24 h or 48 h. As shown in Fig. 4a, b and Figure S4A, chidamide suppressed HL60/ADR cells, K562/A02 cells and THP-1/ADR cells proliferation in a time-dependent manner. And cell viability probability was 43.7% ± 2.0% (HL60/ADR), 42.8% ± 1.4% (K562/A02) and 35.45% ± 1.69% (THP-1/ADR) at a concentration of 2 μM for 48 h. Consistently, when patient-derived R/R AML cells were treated with 0, 0.3, 0.5, 1 μM for 24 h or 48 h, chidamide exhibited potent inhibition in patient-derived anthracycline-resistant AML blasts, especially at 48 h after 1 μM chidamide treatment, which reduced viability to 61.6 ± 6.1% (Fig. 4c). These results suggest that chidamide inhibits the proliferation of anthracycline-resistant AML cells in a dose- and time-dependent manner.

To study whether chidamide induces cell-cycle arrest in resistant AML cells, HL60/ADR cells, K562/A02 and THP-1/ADR cells were treated with 0, 0.01, 0.05, 0.1 μM for 24 h or 48 h, respectively. Flow cytometry was used to measure changes in the cell cycle. Chidamide increased the percentage of cells in G0/G1 phase in a dose- and time-dependent manner (Fig. 4d; Figure S4B; Figure S4D). The percentage of cells in S phase decreased in a dose- and time-dependent manner (Fig. 4e; Figure S4C; Figure S4E).

To determine the apoptotic effect of chidamide on anthracycline-resistant AML cells, HL60/ADR cells, K562/A02 and THP-1/ADR cells were treated with 0, 0.01, 0.05, 0.1 μM for 24 h or 48 h, respectively, and double-stained with Annexin V/PI before being analyzed using flow cytometry. As shown in Fig. 4f-g, Figure S4F-G and Figure S4H-I, chidamide significantly increased apoptotic cells in comparison to control cells. The proportion of apoptotic cells continued to increase following chidamide treatment. These findings suggest that chidamide induces apoptosis in resistant AML cells in a concentration- and time-dependent manner.

To determine the effect of chidamide on anthracycline-sensitive AML cells, the experiments were performed in anthracycline-sensitive HL60, K562 and patient-derived AML cells. CCK-8 assays showed that chidamide inhibited the anthracycline-sensitive AML cell growth. K562 cells, HL60 cells and anthracycline-sensitive primary AML cells, as shown in Figure S4J-L. Cell viability was reduced to 86.5 ± 5.2%(K562, Figure S4J),82.5 ± 2.7%(HL60, Figure S4K) and 88.3 ± 6.6%(primary AML cell, Figure S4L) at a concentration of 0.5 μM for 24 h, and 65.8 ± 2.8%(K562, Figure S4J),63.5 ± 1.8%(HL60, Figure S4K) and 59.1 ± 12.1%(primary AML cell, Figure S4L) for 48 h. Chidamide significantly increased apoptotic cells in comparison to control cells both in K562 cells and HL60 cells (Figure S4M-P), suggesting that chidamide also induced apoptosis in sensitive AML cells. Cell cycle study showed that chidamide increased the rate of cells in G0/G1 phase, while decreased the rate of cells in S phase (Figure S4Q-T). These results demonstrate that chidamide also inhibits the cell viability of anthracycline-sensitive AML cells.

### Combination of chidamide and doxorubicin potently impairs the viability of anthracycline-resistant AML cells

Next, we sought to determine whether chidamide sensitizes anthracycline-resistant AML cells to doxorubicin treatment. As shown in Fig. 4h, i, Figure S4U, and Figure S4V the combination of chidamide and doxorubicin exhibited greater inhibition on the growth of HL60/ADR, K562/A02, THP-1/ADR and patient-derived anthracycline-resistant AML cells, as compared to doxorubicin monotherapy. Inhibitory effects of the combination therapy were significantly greater at 48 h. These data indicate that the inhibitory effects of doxorubicin were significantly enhanced by chidamide in a dose- and time-dependent manner. Therefore, the combination of chidamide and doxorubicin had a pronounced inhibition effect on anthracycline-resistant AML cells.

### Chidamide inhibits HDAC activity in anthracycline-resistant leukemia cells

To test the activity and efficacy of chidamide in anthracycline-resistant AML cells, K562/A02, and patient-derived anthracycline-resistant AML cells were treated with chidamide for 48 h. An HDAC activity assay was used to measure HDAC activity. As shown in Fig. 4j, Figure S4W, HDAC activity was inhibited by chidamide.

### Chidamide suppresses HDAC3-Akt-P21-CDK2 signaling pathways

**Fig. 5:**
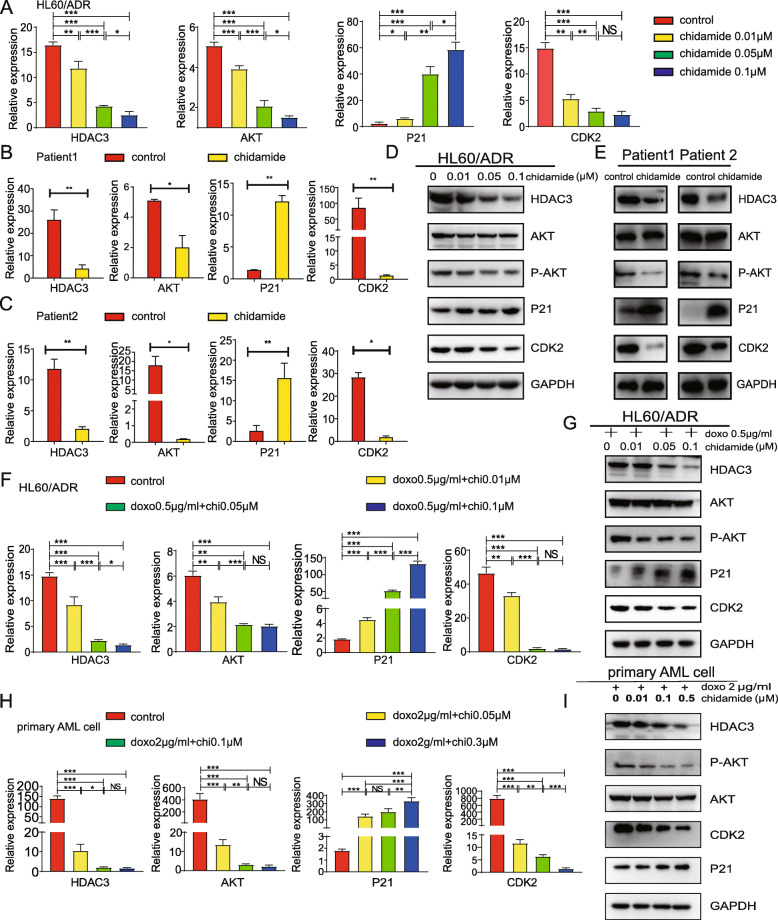
Molecular mechanisms of chidamide activity in HL60/ADR and patient-derived anthracycline-resistant AML cells. Anthracycline-resistant AML cells were treated with different concentrations of chidamide. Expression levels of HDAC3, AKT, P21 and CDK2 were measured using RT-PCR in HL60/ADR (**a**) and primary AML cells (**b**-**c**). **d** HL60/ADR and (**e**) primary AML cells were treated with chidamide and expression levels of HDAC3, P-AKT, AKT, P21, and CDK2 were measured by Western blot. The expression of HDAC3, AKT, P21, and CDK2 was measured by RT-PCR (**f**) and Western blot (**g**) in HL60/ADR cells after treated with a combination of chidamide and doxorubicin. RT-PCR analysis of HDAC3, AKT,CDK2 and P21 (**h**) and western blot analysis (**i**) in primary AML cells treated with combination therapy. GAPDH was used as the loading control. Data represents three independent experiments, and results are shown in the format, means ± S.D. (*P < 0.05, **P < 0.01, ***P < 0.001)

Having shown that HDAC3-AKT-P21-CDK2 signaling pathways were activated in anthracycline-resistant AML cells, we sought to examine whether resistance reversal by chidamide occurs through the HDAC3-AKT-P21-CDK2 signaling pathway. Effects of different doses of chidamide on the HDAC3-AKT-P21-CDK2 pathway were determined by measuring mRNA expression levels in anthracycline-resistant cells. In comparison to control cells of HL60/ADR (Fig. 5a), K562/A02 (Figure S5A) and THP-1/ADR (Figure S5B), chidamide monotherapy led to significant inhibition of the expression of HDAC3, AKT, and CDK2, and increased P21 expression in a dose-dependent manner. Similarly, we treated patient-derived anthracycline-resistant AML cells with 0.3 μM chidamide and found that the expression levels of HDAC3, AKT, and CDK2 decreased while P21 expression increased, compared to control cells (Fig. 5b and c). Furthermore, chidamide inhibited HDAC3, P-AKT, and CDK2 protein expression significantly, but increased P21 protein expression in a dose-dependent manner, while no significant changes in AKT protein levels were observed in HL60/ADR cells, K562/A02 cells, THP-1/ADR and primary AML cells (Fig. 5d, e; Figure S5C-D).

Lastly, HL60/ADR cells, K562/A02 cells, THP-1/ADR and primary AML cells were exposed to a combination of doxorubicin and different doses of chidamide. The results showed that the combination of chidamide and doxorubicin downregulated HDAC3, AKT, and CDK2 expression significantly, while increasing the mRNA levels of P21 in a dose-dependent manner in these anthracycline-resistant cells (Fig. 5f, h; Figure S5E; Figure S5G). Moreover, combination therapy for HL60/ADR cells, K562/A02 cells, THP-1/ADR and primary AML cells decreased HDAC3, P-AKT, CDK2 protein expression, but increased P21 protein expression in a dose-dependent manner (Fig. 5g, i; Figure S5F; Figure S5H). Again, AKT protein levels remained unchanged. These results demonstrate that chidamide monotherapy or the combination therapy of chidamide and doxorubicin are able to suppress the HDAC3-AKT-P21-CDK2 signaling pathway.

### HDAC3 silencing reduces cell proliferation, increases cell apoptosis, induces cell cycle arrest at G0/G1 phase, and suppresses the levels of AKT, P21, and CDK2

**Fig. 6:**
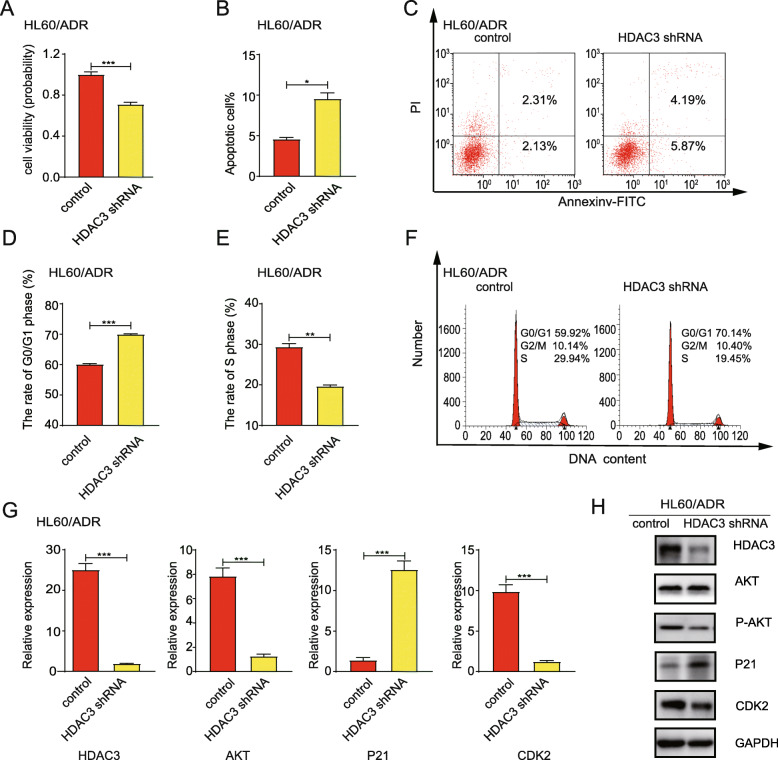
Transfection with HDAC3 shRNA in HL60/ADR cells reduces cell proliferation, increases cell apoptosis, induces cell cycle arrest at G0/G1 phase, and suppresses AKT-P21-CDK2 signaling pathways. **a** CCK-8 assays were used to assess cell proliferation ability. **b** Cell apoptosis was analyzed after HDAC3 knockdown by flow cytometry using Annexin-V/PI staining. **c** is a representative of flow cytometry plot for detection of apoptosis. **d-e** Cell cycles of HL60/ADR cells were analyzed by flow cytometry after PI staining. **f** is a representative of flow cytometry for cell cycle detection. g Expression levels of HDAC3, AKT, P21, and CDK2 were measured by RT-PCR. **h** Expression levels of HDAC3, P-AKT, AKT, P21, and CDK2 were measured by Western blot. GAPDH was used as an internal control. Data represents three independent experiments, and results are shown in the format, mean ± S.D. (*P < 0.05, **P < 0.01, ***P < 0.001, NS: *P* > 0.05)

To examine the effects of HDAC3 silencing on the viability of HL60/ADR cells and K562/A02 cells, HL60/ADR cells and K562/A02 cells were transfected with HDAC3 shRNA. CCK-8 assays showed that cell proliferation was significantly suppressed by 71.08 ± 1.92% (HL60/ADR cells), 50.5 ± 2.6%(K562/A02 cells) in HDAC3 shRNA knockdown cells (Fig. 6a; Figure S6A). The proportion of apoptotic cells was greater in cells with HDAC3 knockdown compared to control cells (Fig. 6b-c; Figure S6B-C). Moreover, HDAC3 silencing reduced the proportion of cells in the S phase and induced cell cycle arrest in G0/G1 phase (Fig. 6d-f; Figure S6D-F).

Next, we analyzed the effect of HDAC3 silencing on AKT-P21-CDK2 signal axis by qRT-PCR and Western blot. As anticipated, transfection with HDAC3 shRNA resulted in a decrease in HDAC3, AKT, and CDK2 expression, but an increase in mRNA and protein levels of P21 (Fig. 6g-h; Figure S6G-H). These results further support the hypothesis that HDAC3 regulates the activity of AKT. Inhibiting HDAC3 expression decreases the expression of P-AKT and inactivates the AKT-P21-CDK2 signal axis.

### AKT inhibitor reduces cell proliferation, increases cell apoptosis, induces cell cycle arrest at G0/G1 phase, and suppresses CDK2-P21 signaling pathways

**Fig. 7:**
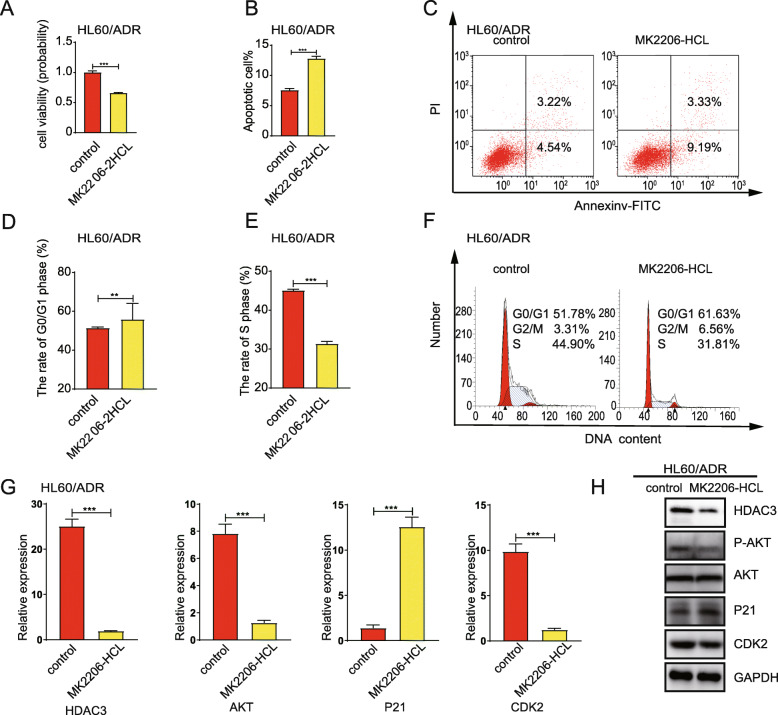
AKT inhibitor reduces cell proliferation, increases cell apoptosis, induces cell cycle arrest at G0/G1 phase, and suppresses the expression of HDAC3, CDK2. HL60/ADR cells were incubated with an AKT inhibitor (MK2206-2HCL). (**a**) CCK-8 assays were used to assess cell proliferation ability. (**b**) Flow cytometry was used to analyze cell apoptosis. **c** Representative of flow cytometry plot for detection of apoptosis. **d-e** Cell cycles of HL60/ADR cells were analyzed by flow cytometry after PI staining. **f** is a representative of flow cytometry for cell cycle detection. **g** Expression levels of HDAC3, AKT, P21, and CDK2 were measured by RT-PCR. **h** Expression levels of HDAC3, P-AKT, AKT, P21, and CDK2 were measured by Western blot. GAPDH was used as an internal control. Data represents three independent experiments, results are shown in the format, mean ± S.D. (*P < 0.05, **P < 0.01, ***P < 0.001, NS: *P* > 0.05)

To determine the role of AKT in drug resistance, HL60/ADR cells and K562/A02 cells were exposed to MK2206-HCL, an AKT inhibitor. CCK-8 assays revealed that MK2206-HCL treatment significantly reduced cell proliferation to 66.04 ± 0.63% (HL60/ADR cells, Fig. 7a), 29.0 ± 11.3% (K562/A02 cells, Figure S7A). Moreover, AKT inhibition led to a higher ratio of apoptotic cells (Fig. 7b-c; Figure S7B-C), a reduction of cell proportion in S phase, as well as an induction of cell cycle arrest at the G0/G1 phase (Fig. 7d-f; Figure S7D-F). Taking the mechanisms into account, treatment with AKT inhibitor resulted in the mRNA and protein expression decrease of HDAC3 and CDK2 (Fig. 7g-h; Figure S7G-H).

### Suppression of HDAC3 and AKT are required for chidamide induced anthracycline-resistant AML cells inhibition

**Fig. 8:**
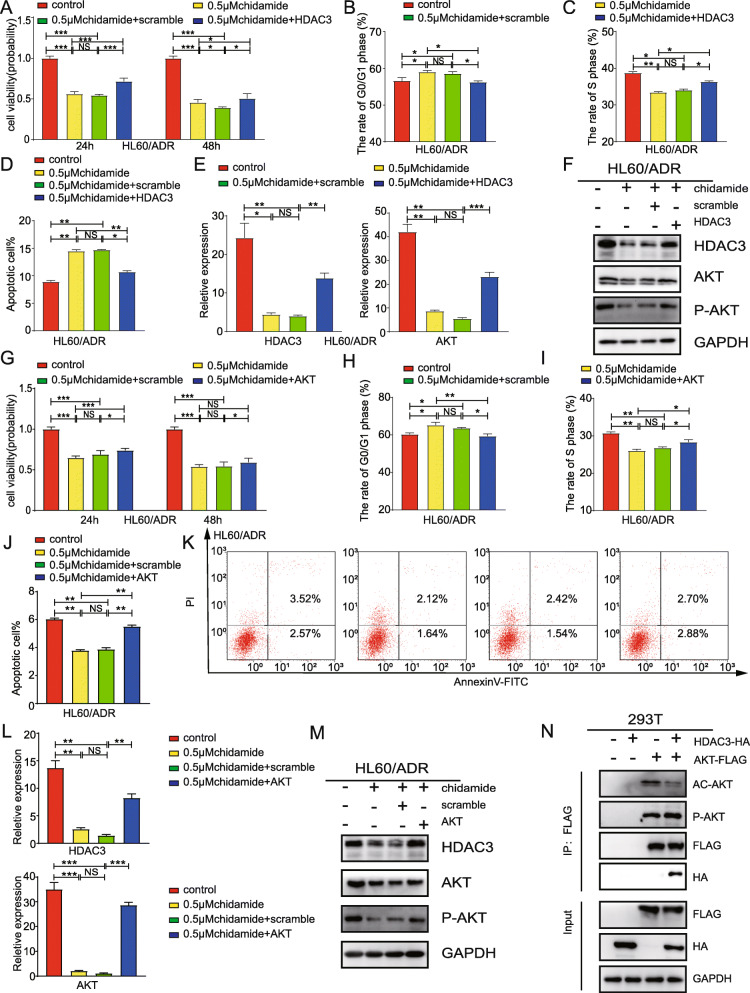
Suppression of HDAC3 and AKT are required for chidamide induced anthracycline-resistant AML cells inhibition. HL60/ADR cells were treated with chidamide for 48 h and transfected with overexpressed HDAC3 plasmids or control plasmids. **a** CCK-8 assays were used to assess cell proliferation ability. **b-c** Cell cycle detection by flow cytometry. **d** Detection of apoptosis by flow cytometry. The expression of HDAC3 and AKT was measured by RT-PCR (**e**) and western blot analysis (**f**) HL60/ADR cells were treated with chidamide for 48 h and transfected with overexpressed AKT plasmids or control plasmids. **g** CCK-8 assays were used to assess cell proliferation ability. **h-i** Cell cycle detection by flow cytometry. **j-k** Detection of apoptosis by flow cytometry. The expression of HDAC3 and AKT was measured by RT-PCR **(l)** and western blot analysis (**m**). **n** Co-immunoprecipitation of AKT and HDAC3 were performed in 293 T cells. Data represents three independent experiments, data are expressed as mean values± S.D. (*P < 0.05, **P < 0.01, ***P < 0.001, NS: *P* > 0.05)

To investigate whether chidamide-induced anthracycline-resistant AML cells inhibition was associated with the inactivation of HDAC3 in an AKT-dependent manner, we overexpressed HDAC3 or AKT after chidamide-induced leukemia inhibition. We found that overexpression of HDAC3 rescued the decreased cell viability (Fig. 8a), relieved cell cycle arrest (Fig. 8b-c) and reduced the rate of apoptosis cells (Fig. 8d) induced by chidamide. In addition, overexpression of HDAC3 upregulated the chidamide-decreased AKT at mRNA level, and increased AKT phosphorylation (Fig. 8e-f). Silimarly, overexpression of AKT also restored cell viability (Fig. 8g), relieved cell cycle arrest (Fig. 8h-i) and reduced the rate of apoptosis cells (Fig. 8j-k).

### HDAC3 binds AKT and increases the AKT phosphorylation while activated AKT contributes to HDAC3 expression

KC Hung had reported that activated AKT upregulated HDAC3 expression [26]. And we also showed that overexpression of AKT increased the mRNA and protein level of HDAC3 (Fig. 8l-m). To elucidate the mechanism underlying HDAC3 and AKT circuit and whether HDAC3 is responsible for AKT phosphorylation, we performed co-immunoprecipitation to verify the HDAC3-AKT interaction in HEK293T cells coexpressing HDAC3 and AKT. Co-immunoprecipitation demonstrated protein interaction of HDAC3 and AKT. The results of Western blotting with anti-acetylation and phosphorylation revealed a higher level of phosphorylation and lower acetylation in cells coexpressing HDAC3 and AKT compared to that in cells expressing AKT alone, which might result from endogenous HDAC3 (Fig. 8n). These results support the idea that HDAC3 binds AKT and increases the AKT phospharylation.

## Discussion

Currently, the treatment for relapsed/refractory AML remains a major challenge. A drug or treatment regimen is required to improve the remission rates for R/R to prolong overall survival time. It is necessary to discover the mechanism behind AML resistance, and find target drugs or regimens to overcome drug resistance, so as to improve the CR rate of patients with relapsed/refractory AML, prolong disease-free survival time and overall survival rates.

This study shown that the chidamide-based regimen improves the overall remision rate of patients with relapsed/refractory AML, so we want to further analyze the mechanism of bring such good clinical therapeutic results. To obtain a better understanding of the mechanism of drug resistance, we performed RNA-sequencing in drug-resistant and control cells. From the results of RNA-sequencing, we found that the PI3K-AKT signaling pathway was activated and HDAC3 was upregulated in drug-resistant cells.

Activated AKT regulated apoptosis, cell growth, and the cell-cycle through phosphorylation of numerous downstream targets [27]. Inhibition of the PI3K-AKT signaling pathway decreased leukemia stem cell growth and increased apoptosis [28, 29]. Moreover, the PI3K/AKT signaling pathway is often hyperactive in AML patients with poor prognoses [28–30]. In several types of cancer, including AML, activation of the PI3K/AKT signaling pathway reduces sensitivity to chemotherapeutic drugs [31].

It was shown that the aberrant recruitment of HDACs plays an important role in leukemogenesis. Changes in activity and/or expression levels of HDACs were also observed in leukemia and solid tumors [32, 33]. Overexpression of HDACs in tumor cells was shown to protect cells from genotoxic insults [23]. For example, the expression of HDAC3 was shown to be vital to the maintenance of genome stability and DNA damage control. HDAC3 knockdown impaired DNA repair [34] and resulted in growth inhibition of human colon cancer cell lines [35]. Moreover, depletion or pharmacological inhibition of HDAC3 triggered apoptosis in cutaneous T-cell lymphoma and multiple myeloma [36, 37].

Previous studies have confirmed the close relationship between HDAC3 and AKT [24, 25]. HDAC3 binds to AKT in several cell lines, and regulates the phosphorylation and acetylation levels of AKT. Overexpression of HDAC3 reduces AKT acetylation levels but increases AKT phosphorylation levels.

Taking earlier research and this study into account, it is evident that HDAC3 and P-AKT expressions are positively correlated in drug-resistant K562/A02 cells. In addition, activation of the HDAC3-AKT-P21-CDK2 pathway represents one of the mechanisms through which K562/A02 cells achieve resistance to anthracycline. Therefore, there is an urgent need for a drug to inhibit this signaling pathway and reverse drug resistance. Here, we found that chidamide inhibits cell proliferation, increases cell apoptosis, and induces cell-cycle arrest in a time- and dose-dependent manner in anthracycline-resistant AML cells. These effects are associated with the inhibition of the HDAC3 -AKT-P21-CDK2 signaling pathway.

Our study showed that expression levels of HDAC3 had increased in anthracycline-resistant cells. Univariate and multivariate analyses confirmed that HDAC3 had an adverse effect on OS and EFS. Our research showed that the HDAC inhibitor, chidamide also exhibited a significant inhibitory effect on anthracycline-resistant AML cell growth by suppressing HDAC3 expression. This result is consistent with another study that showed that chidamide inhibits pancreatic tumor growth by suppressing the expression of HDACs [38].

Cyclin-dependent kinases (CDKs) are a family of serine/threonine protein kinases that regulate cell cycle progression [39]. CDK2 activity is necessary for cells to progress through the S phase [40, 41]. CDKs are inhibited by CDK inhibitors (CKIs) [42], such as P21, which suppresses CDK2 activity and blocks cell cycle transition from G1 phase to S phase [43–45]. Moreover, the expression of P21 has always been regulated by HDAC inhibitors [38, 46, 47] and P21 is upregulated following HDAC3 depletion [48]. In this study, the HDAC inhibitor, chidamide induced cell-cycle arrest in a time- and dose-dependent manner in anthracycline-resistant AML cells. The mechanism of action may be through the inhibition of CDK2 expression and increase in P21 expression.

Patients who are resistant to primary chemotherapy or have relapsed have poor prognoses due to the inability to control disease progression and therapeutic complications. Histone deacetylases (HDACs) are responsible for the regulation of gene transcription, protein function, and stability [49, 50]. HDAC inhibitors are able to potently induce cell cycle arrest, differentiation, and apoptosis of malignant cells [51]. HDAC inhibitors have been used in the treatment of several hematologic tumors, such as acute myeloid leukemia and T-cell lymphomas [52–62]. In the present study, 27 patients who experienced R/R after receiving anthracycline therapy were given a combination of chidamide and anthracycline-based regimen. The combination of chidamide and anthracycline increases the sensitivity of leukemia cells to anthracyclines and is able to reverse drug resistance.

## Conclusion

In summary, our study showed that The combination of chidamide and anthracycline-based regimen is a promising treatment for R/R patients. The abnormality of the HDAC3-AKT-P21-CDK2 signaling pathway may be one of the mechanisms through which AML cells achieve anthracycline resistance. Chidamide inhibits cell proliferation, as well as induces cell-cycle arrest and apoptosis in a time- and dose-dependent manner in R/R AML cells. These effects are associated with an inhibition of the HDAC3-AKT-P21-CDK2 pathway.

## Supplementary Information

**Additional file 1: Supplemental Figure 1**. Anthracycline-resistant cell lines HL60/ADR cells, K562/A02 cells and THP/ADR cells showed resistance to doxorubicin. Cell viability was examined after treatment with different doses of doxorubicin for 24 h by CCK-8 assay. **(A)** HL60, **(B)** HL60/ADR, **(C)** K562, **(D)** K562/A02, **(E)** THP-1, **(F)** THP-1/ADR. **Supplemental Figure 2**. Differential gene and protein expression in K562 and K562/A02. **(A)** RT-PCR analysis showed differential gene expression in K562 and K562/A02. **(B)** Western blot analysis shows protein expression of differential gene in K562 and K562/A02. Data represent three independent experiments, results are shown in the format, mean ± S.D. (*P < 0.05, **P < 0.01, ***P < 0.001, NS: *P* > 0.05). **Supplemental Figure 3**. Chidamide sensitizes anthracycline-resistant cells to anthracycline in vivo. **(A)** K562 cells (1 × 10^7^ cells) were implanted into NOD/SCID mice. The mice were randomly divided into 4 groups (5 mice in each group). Doxorubicin was injected into the mice at day 5 and day 12 after leukemic cells inoculation. And chidamide was administered 3 times every week from day 6 after leukemic cells inoculation. (B) The volume of each tumor was measured every 3 days. The tumor volume was calculated by the formula (V = 0.5*length*width^2^). (C) The visual analysis of tumors harvested from mice. (D-E) The measurement of xenograft tumor volume and weight. (F) The mRNA expression levels of HDAC3, AKT, P21 and CDK2 were measured using RT-PCR in tumor sections. Data represent three independent experiments, data are expressed as mean values± S.D. (*P < 0.05, **P < 0.01, ***P < 0.001, NS: *P* > 0.05). **Supplemental Figure 4A-I**. Chidamide affected the behavior of anthracycline- resistant cells. **(A)** CCK-8 assays were used to assess the proliferative abilities of K562/A02 cells. The proportion of G0/G1 phase **(B)** and S phase **(C)** were measured in response to incubation with chidamide in K562/A02 cells. The proportion of G0/G1 phase **(D)** and S phase **(E)** were measured in THP-1/ADR cells. **(F-G)** represent apoptosis analysis with flow cytometry in K562/A02 cells and **(H-I)** in THP-1/ADR cells (Annexin V/PI). Data represent three independent experiments, results are shown in the format, mean ± S.D. (*P < 0.05, **P < 0.01, ***P < 0.001, NS: *P* > 0.05). **Supplemental Figure 4J-W. K562**, HL60 and patient-derived anthracycline-sensitive AML cells are sensitive to chidamide monotherapy, and chidamide sensitizes anthracycline-resistant cells to anthracycline in K562/A02 and THP/ADR cells. CCK-8 assays were used to assess the proliferative abilities of K562 **(J)**, HL60 **(K)**, patient-derived cells **(L)**. **(M-N)** are representatives of flow cytometry (Annexin V/PI) for detection of apoptosis. The apoptotic rate of cells was measured by flow cytometry in K562 **(O)** and HL60 cells **(P)**. **(Q-R)** represent cell cycle analysis with flow cytometry in K562 cells and HL60 cells. The proportion of G0/G1 phase and S phase were measured in response to incubation with chidamide in K562 cells **(S)** and HL60 cells **(T)**. The cell viability of **(U)** K562/A02 and **(V)** THP-1/ADR cells treated by combination therapy were measured with CCK-8 assays. **(W)** HDAC activity of K562/A02 cells was measured after treated with chidamide. Data represent three independent experiments, results are shown in the format, mean ± S.D. (*P < 0.05, **P < 0.01, ***P < 0.001, NS: *P* > 0.05). **Supplemental Figure 5**. Molecular mechanisms of chidamide activity in K562/A02 and THP-1/ADR cells. Anthracycline-resistant AML cells were treated with different concentrations of chidamide. Expression levels of HDAC3, AKT, P21 and CDK2 were measured using RT-PCR in K562/A02 cells **(A)** and THP-1/ADR cells **(B). (C)** K562/A02 and **(D)** THP-1/ADR were treated with chidamide and the expression levels of HDAC3, P-AKT, AKT, P21 and CDK2 were measured by Western blot. RT-PCR analysis of HDAC3, AKT, CDK2 and P21 **(E)** and western blot analysis **(F)** in K562/ADR cells treated with a combination of chidamide and doxorubicin. The expression of pathway genes was measured by RT-PCR **(G)** and Western blot **(H)** in THP-1/ADR cells treated with combination therapy. Data represent three independent experiments, and results are shown in the format, means ± S.D. (*P < 0.05, **P < 0.01, ***P < 0.001). **Figure S6.** Transfection with HDAC3 shRNA in K562/A02 cells reduces cell proliferation, increases cell apoptosis, induces cell cycle arrest at G0/G1 phase, and suppresses AKT-P21-CDK2 signaling pathways. **(A)** CCK-8 assays were used to assess cell proliferation ability. **(B)** Cell apoptosis was analyzed after HDAC3 knockdown by flow cytometry using Annexin-V/PI staining. **(C)** is a representative of flow cytometry plot for detection of apoptosis. **(D-E)** Cell cycles of K562/A02 cells were analyzed by flow cytometry after PI staining. **(F)** is a representative of flow cytometry for cell cycle detection. **(G)** Expression levels of HDAC3, AKT, P21 and CDK2 were measured by RT-PCR. **(H)** Expression levels of HDAC3, P-AKT, AKT, P21 and CDK2 were measured by Western blot. GAPDH was used as an internal control. Data represent three independent experiments, and results are shown in the format, mean ± S.D. (*P < 0.05, **P < 0.01, ***P < 0.001, NS: *P* > 0.05). **Figure S7**. AKT inhibitor reduces cell proliferation, increases cell apoptosis, induces cell cycle arrest at G0/G1 phase, and suppresses the expression of HDAC3, CDK2 and P21. K562/A02 cells were incubated with an AKT inhibitor (MK2206-2HCL). **(A)** CCK-8 assays were used to assess cell proliferation ability. **(B)** Flow cytometry was used to analyze cell apoptosis. **(C)** is a representative of flow cytometry plot for detection of apoptosis. **(D-E)** Cell cycles of K562/A02 cells were analyzed by flow cytometry after PI staining. **(F)** is a representative of flow cytometry for cell cycle detection. **(G)** Expression levels of HDAC3, AKT, P21 and CDK2 were measured by RT-PCR. **(H)** Expression levels of HDAC3, P-AKT, AKT, P21 and CDK2 were measured by Western blot. GAPDH was used as an internal control. Data represent three independent experiments, results are shown in the format, mean ± S.D. (*P < 0.05, **P < 0.01, ***P < 0.001, NS: *P* > 0.05). **Table S1.**Comparison of different anthracycline-based chemotherapy regimens.

## Data Availability

All data generated or analyzed during this study are included in this published article.

## Abbreviations

AML: Acute myeloid leukemia
R/R: Relapse or refractory
EFS: Event free survival
OS: Overall survival
HDAC3: Histone deacetylase 3
AKT: AKT serine/threonine kinase 1
P21: P21 calcium binding protein
CDK2: Cyclin dependent kinase 2
PI3K: Phosphoinositide 3-kinase
CR: Complete remission
HDAC: Histone deacetylase
NCCN: The National Comprehensive Cancer Network
DAC: Decitabine
Acla: Aclarubicin
Arac: Cytarabine
G-CSF: Granulocyte colony stimulating factor
VP: Vincristine and prednisone
RPKM: Reads per kilobase per million mapped reads
TCGA: The Cancer Genome Atlas database
ATCC: America type culture collection
RPMI 1640: Roswell Park Memorial Institute 1640
DMEM: Dulbecco’s modified eagle medium
FBS: Fetal bovine serum
PBMCs: Peripheral blood mononuclear cells
CCK-8: Cell Counting Kit-8
PI: Propidium Iodide
FITC: Fluorescein Isothiocyanate
RT-PCR: Real time polymerase chain reaction
SDS-PAGE: Sodium dodecyl sulfate polyacrylamide gel electrophoresis
NOD/SCID: Nod-obese diabetic/severecombined immunodeficiency disease
KEGG: Kyoto Encyclopedia of Genes and Genomes
GO: Gene ontology
FLT3/ITD: Fms-like tyrosine kinase internal tandem duplication
DNMT3A: Recombinant DNA methyltransferase 3A
BCOR: BCL6 corepressor
NRAS: N-ras oncogene
STAG2: Stromal antigen 2
PFS: Progression-free survival
WBC: White blood cell
Doxo: Doxorubicin
Chi: Chidamide
