# Supplementary material for: Chidamide increases the sensitivity of refractory or relapsed acute myeloid leukemia cells to anthracyclines via regulation of the HDAC3 -AKT-P21-CDK2 signaling pathway
Source: J Exp Clin Cancer Res. 2020 Dec 9;39:278. doi: 10.1186/s13046-020-01792-8 (PMC7724824; doi:10.1186/s13046-020-01792-8)
Supplement: Supplementary file 1 — Additional file 1: Supplemental Figure 1. Anthracycline-resistant cell lines HL60/ADR cells, K562/A02 cells and THP/ADR cells showed resistance to doxorubicin. Cell viability was examined after treatment with different doses of doxorubicin for 24 h by CCK-8 assay. (A) HL60, (B) HL60/ADR, (C) K562, (D) K562/A02, (E) THP-1, (F) THP-1/ADR. Supplemental Figure 2. Differential gene and protein expression in K562 and K562/A02. (A) RT-PCR analysis showed differential gene expression in K562 and K562/A02. (B) Western blot analysis shows protein expression of differential gene in K562 and K562/A02. Data represent three independent experiments, results are shown in the format, mean ± S.D. (*P < 0.05, **P < 0.01, ***P < 0.001, NS: P > 0.05). Supplemental Figure 3. Chidamide sensitizes anthracycline-resistant cells to anthracycline in vivo. (A) K562 cells (1 × 10^7^ cells) were implanted into NOD/SCID mice. The mice were randomly divided into 4 groups (5 mice in each group). Doxorubicin was injected into the mice at day 5 and day 12 after leukemic cells inoculation. And chidamide was administered 3 times every week from day 6 after leukemic cells inoculation. (B) The volume of each tumor was measured every 3 days. The tumor volume was calculated by the formula (V = 0.5*length*width2). (C) The visual analysis of tumors harvested from mice. (D-E) The measurement of xenograft tumor volume and weight. (F) The mRNA expression levels of HDAC3, AKT, P21 and CDK2 were measured using RT-PCR in tumor sections. Data represent three independent experiments, data are expressed as mean values± S.D. (*P < 0.05, **P < 0.01, ***P < 0.001, NS: P > 0.05). Supplemental Figure 4A-I. Chidamide affected the behavior of anthracycline- resistant cells. (A) CCK-8 assays were used to assess the proliferative abilities of K562/A02 cells. The proportion of G0/G1 phase (B) and S phase (C) were measured in response to incubation with chidamide in K562/A02 cells. The proportion of G0/G1 phase (D) and S phase (E [file 13046_2020_1792_MOESM1_ESM.docx]

**SUPPLEMENTARY INFORMATION**

**Supplementary Figures and Figure Legends**

**
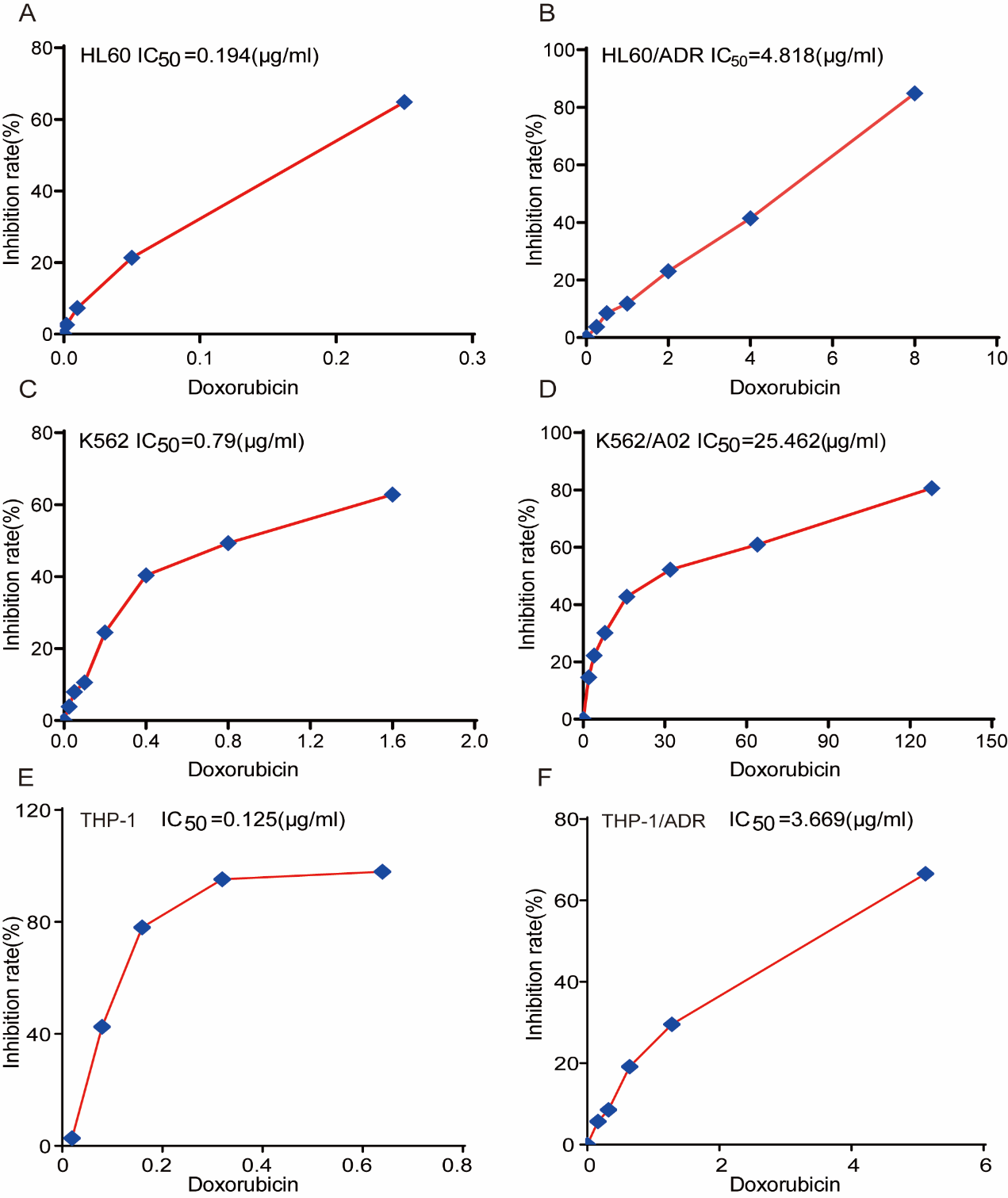
**

**Supplemental Figure 1. Anthracycline-resistant cell lines HL60/ADR cells，K562/A02 cells and THP/ADR cells showed resistance to doxorubicin. Cell viability was examined after treatment with different doses of doxorubicin for 24 h by CCK-8 assay. (A)** HL60, **(B)** HL60/ADR, **(C)** K562, **(D)** K562/A02, **(E)** THP-1, **(F)** THP-1/ADR.


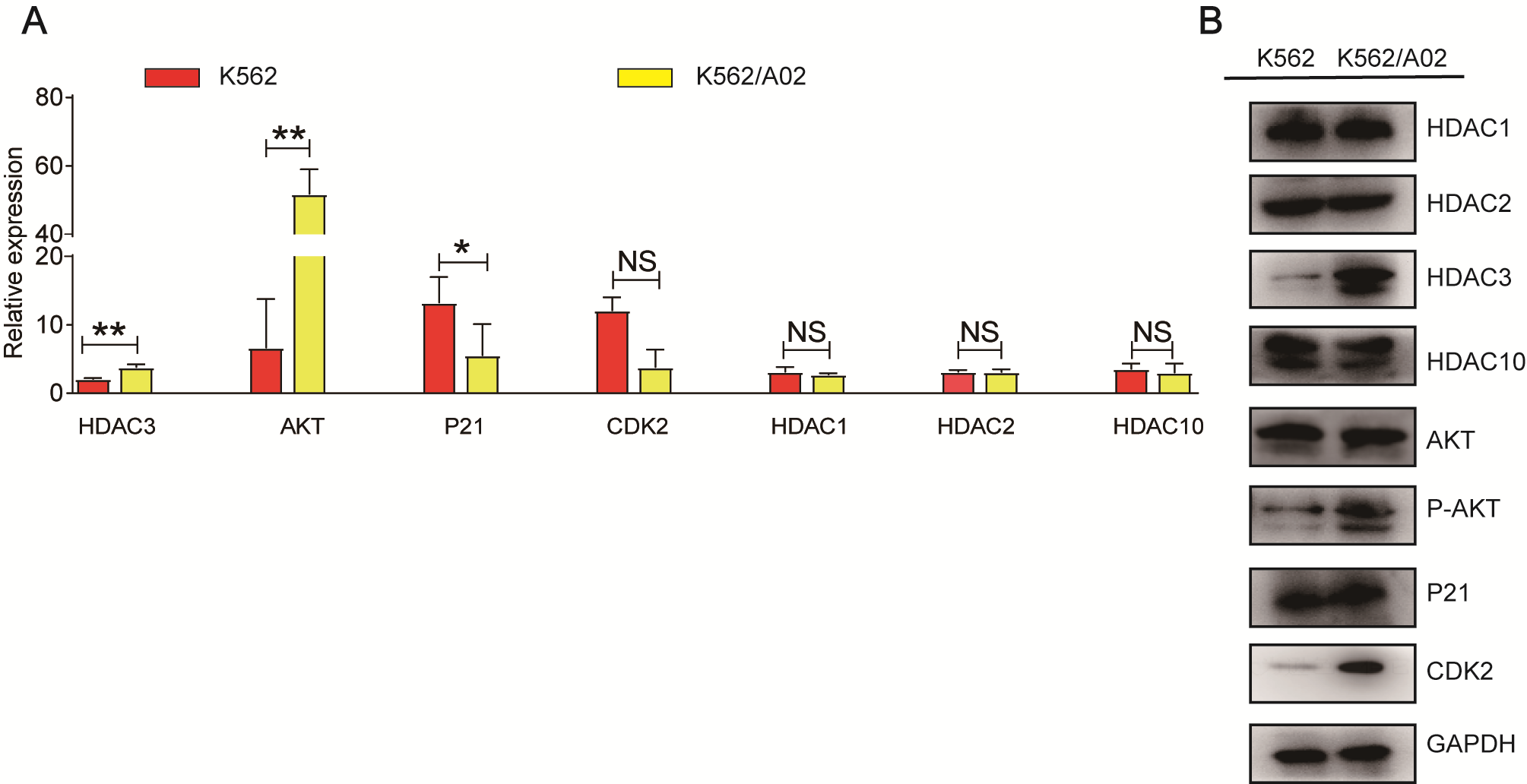


**Supplemental Figure 2. Differential gene and protein expression in K562 and K562/A02.** **(A)** RT-PCR analysis showed differential gene expression in K562 and K562/A02. **(B)** Western blot analysis shows protein expression of differential gene in K562 and K562/A02. Data represent three independent experiments, results are shown in the format, mean ± S.D. (**P* < 0.05, ***P* < 0.01, ****P* < 0.001, NS: *P* > 0.05).


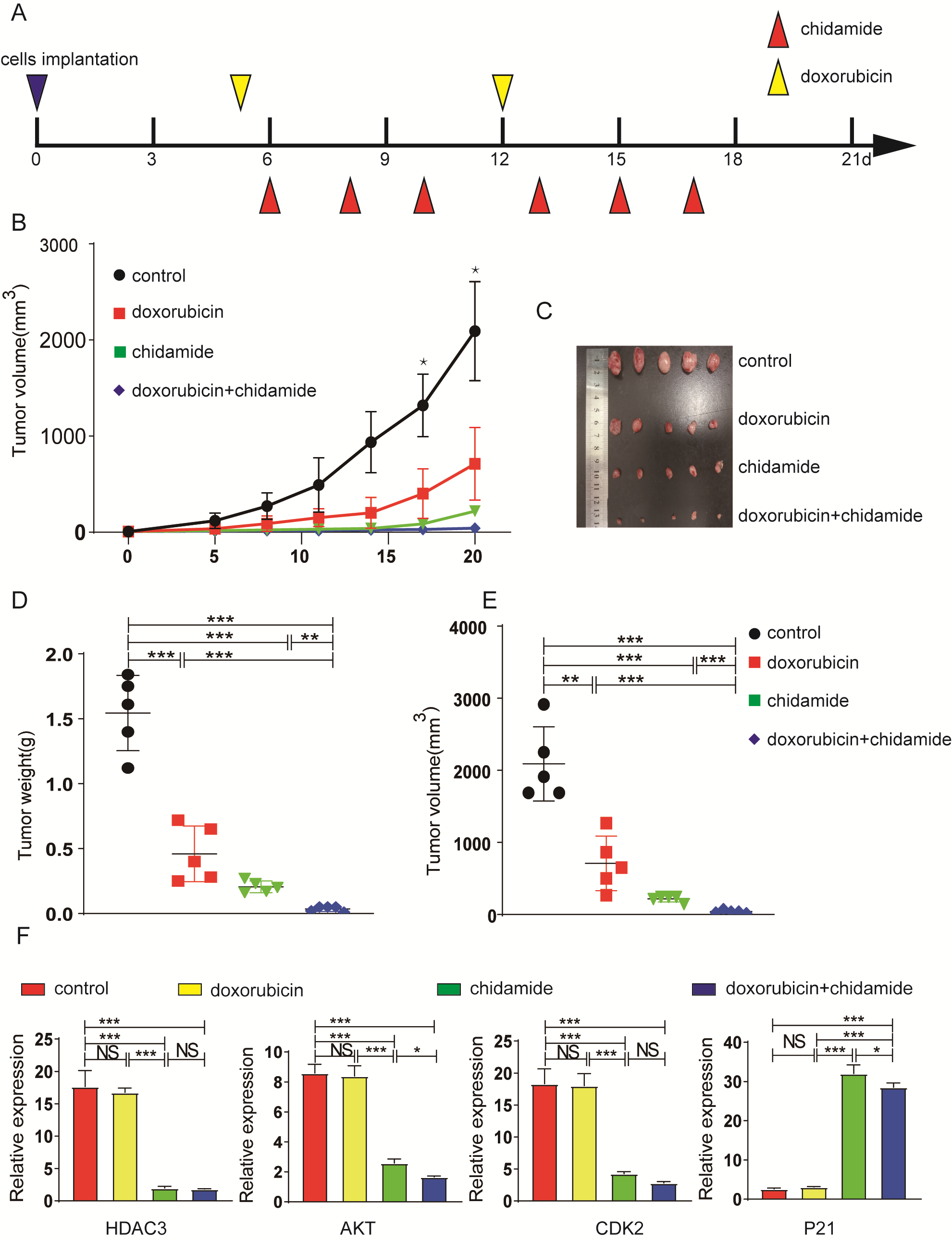


**Supplemental Figure 3. Chidamide sensitizes anthracycline-resistant cells to anthracycline in vivo. (A)** K562 cells (1 × 10^7^ cells) were implanted into NOD/SCID mice. The mice were randomly divided into 4 groups (5 mice in each group). Doxorubicin was injected into the mice at day 5 and day 12 after leukemic cells inoculation. And chidamide was administered 3 times every week from day 6 after leukemic cells inoculation. (B) The volume of each tumor was measured every 3 days. The tumor volume was calculated by the formula (V=0.5*length*width^2^). (C) The visual analysis of tumors harvested from mice. (D-E) The measurement of xenograft tumor volume and weight. (F) The mRNA expression levels of HDAC3, AKT, P21 and CDK2 were measured using RT-PCR in tumor sections. Data represent three independent experiments, data are expressed as mean values± S.D. (*P < 0.05, **P < 0.01, ***P < 0.001, NS: P > 0.05).


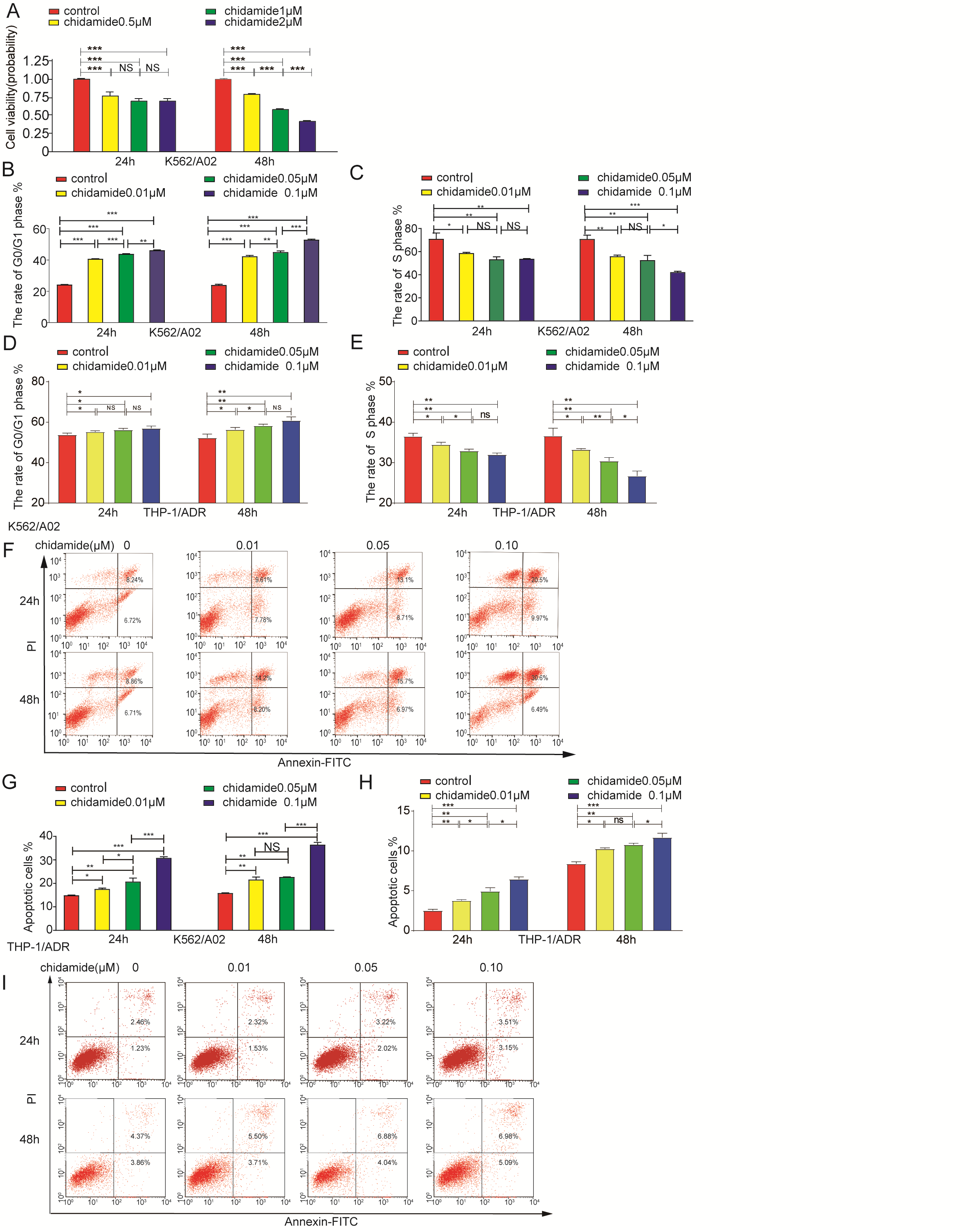


**Supplemental Figure 4A-I. Chidamide affected the behavior of anthracycline- resistant cells. (A)** CCK-8 assays were used to assess the proliferative abilities of K562/A02 cells. The proportion of G0/G1 phase **(B)** and S phase **(C)** were measured in response to incubation with chidamide in K562/A02 cells. The proportion of G0/G1 phase **(D)** and S phase **(E)** were measured in THP-1/ADR cells. **(F-G)** represent apoptosis analysis with flow cytometry in K562/A02 cells and **(H-I)** in THP-1/ADR cells (Annexin V/PI). Data represent three independent experiments, results are shown in the format, mean ± S.D. (**P* < 0.05, ***P* < 0.01, ****P* < 0.001, NS: *P* > 0.05).


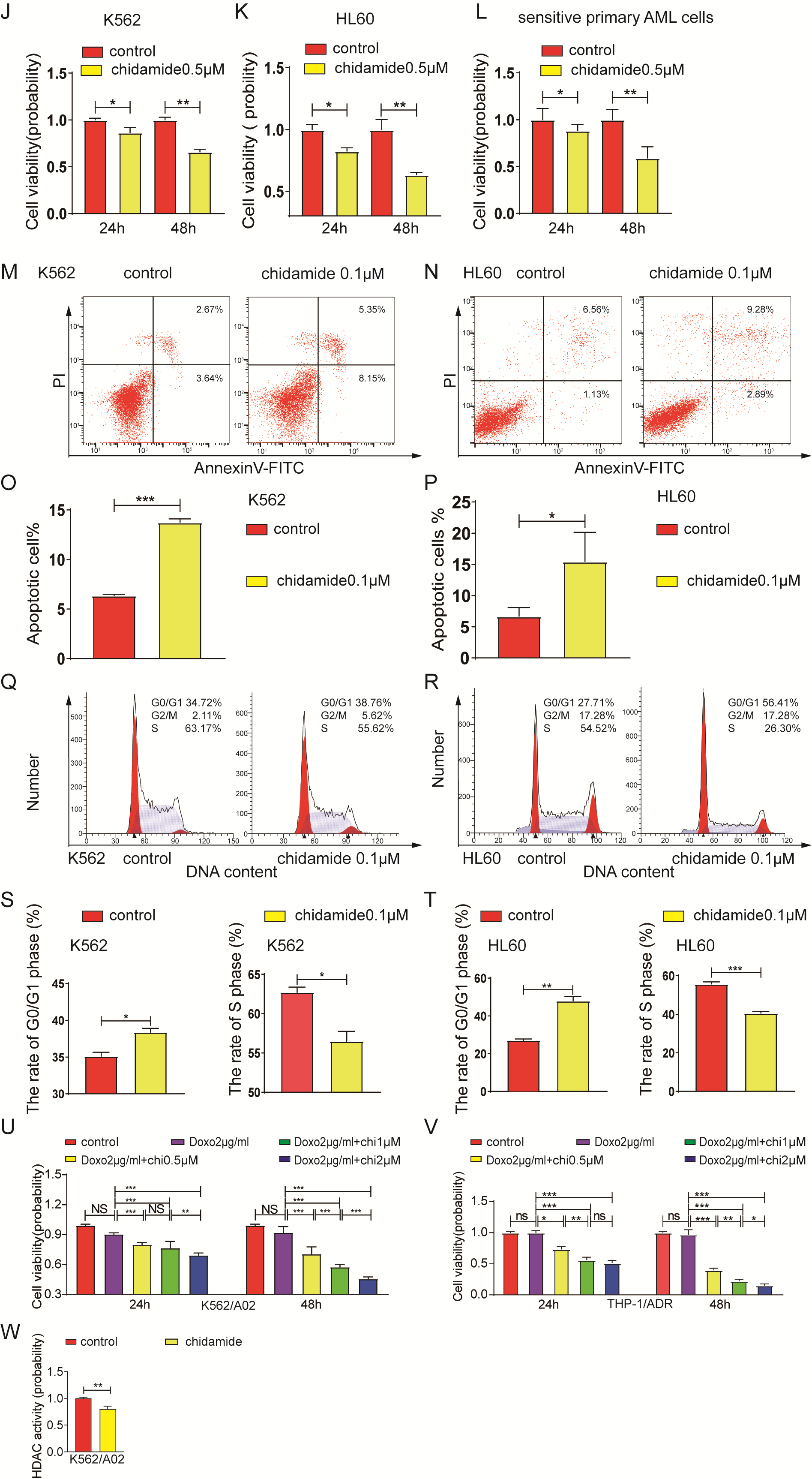


**Supplemental Figure 4J-W. K562, HL60 and patient-derived anthracycline-sensitive AML cells are sensitive to chidamide monotherapy, and chidamide sensitizes anthracycline-resistant cells to anthracycline in K562/A02 and THP/ADR cells.** CCK-8 assays were used to assess the proliferative abilities of K562 **(J)**, HL60 **(K)**, patient-derived cells **(L)**. **(M-N)** are representatives of flow cytometry (Annexin V/PI) for detection of apoptosis. The apoptotic rate of cells was measured by flow cytometry in K562 **(O)** and HL60 cells **(P)**. **(Q-R)** represent cell cycle analysis with flow cytometry in K562 cells and HL60 cells. The proportion of G0/G1 phase and S phase were measured in response to incubation with chidamide in K562 cells **(S)** and HL60 cells **(T)**. The cell viability of **(U)** K562/A02 and **(V)** THP-1/ADR cells treated by combination therapy were measured with CCK-8 assays. **(W)** HDAC activity of K562/A02 cells was measured after treated with chidamide. Data represent three independent experiments, results are shown in the format, mean ± S.D. (**P* < 0.05, ***P* < 0.01, ****P* < 0.001, NS: *P* > 0.05).


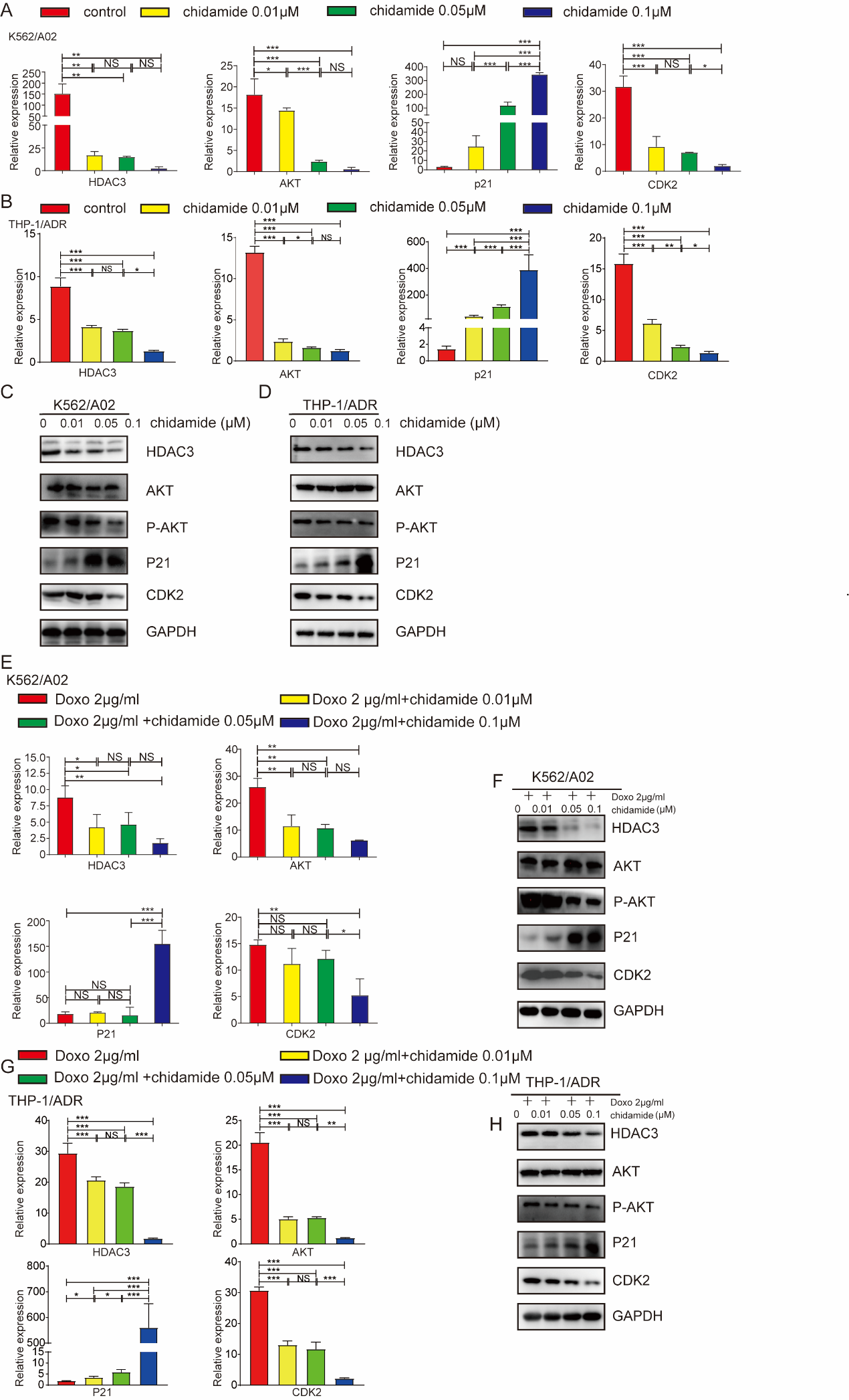


**Supplemental Figure 5. Molecular mechanisms of chidamide activity in K562/A02 and THP-1/ADR cells.** Anthracycline-resistant AML cells were treated with different concentrations of chidamide. Expression levels of HDAC3, AKT, P21 and CDK2 were measured using RT-PCR in K562/A02 cells **(A)** and THP-1/ADR cells **(B). (C)** K562/A02 and **(D)** THP-1/ADR were treated with chidamide and the expression levels of HDAC3, P-AKT, AKT, P21 and CDK2 were measured by Western blot. RT-PCR analysis of HDAC3, AKT, CDK2 and P21 **(E)** and western blot analysis **(F)** in K562/ADR cells treated with a combination of chidamide and doxorubicin. The expression of pathway genes was measured by RT-PCR **(G)** and Western blot **(H)** in THP-1/ADR cells treated with combination therapy. Data represent three independent experiments, and results are shown in the format, means ± S.D. (**P* < 0.05, ***P* < 0.01, ****P* < 0.001).

**
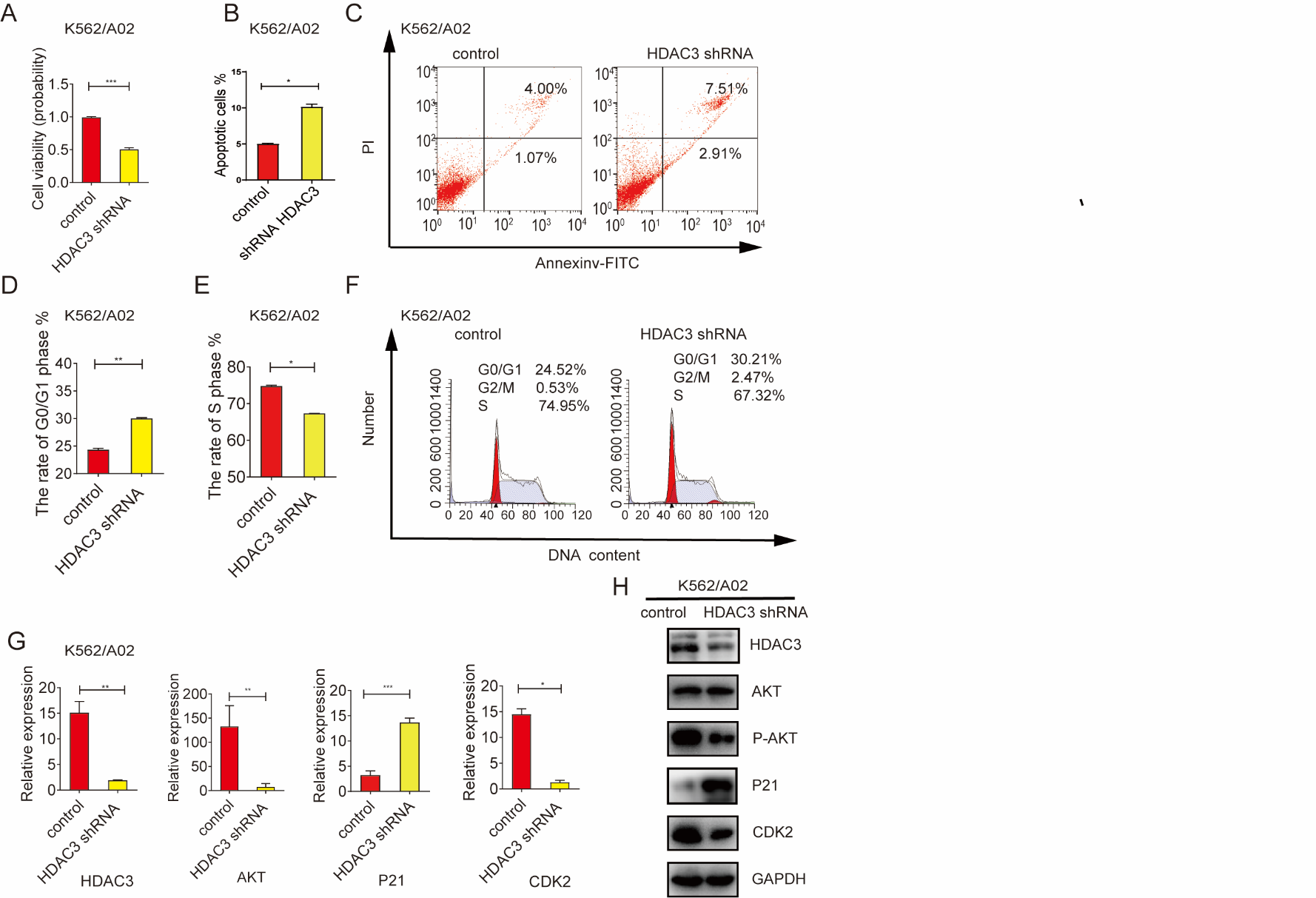
**

**Figure S6. Transfection with HDAC3 shRNA in K562/A02 cells reduces cell proliferation, increases cell apoptosis, induces cell cycle arrest at G0/G1 phase, and suppresses AKT-P21-CDK2 signaling pathways. (A)** CCK-8 assays were used to assess cell proliferation ability. **(B)** Cell apoptosis was analyzed after HDAC3 knockdown by ﬂow cytometry using Annexin-V/PI staining. **(C)** is a representative of flow cytometry plot for detection of apoptosis. **(D-E)** Cell cycles of K562/A02 cells were analyzed by ﬂow cytometry after PI staining. **(F)** is a representative of flow cytometry for cell cycle detection. **(G)** Expression levels of HDAC3, AKT, P21 and CDK2 were measured by RT-PCR. **(H)** Expression levels of HDAC3, P-AKT, AKT, P21 and CDK2 were measured by Western blot. GAPDH was used as an internal control. Data represent three independent experiments, and results are shown in the format, mean ± S.D. (**P* < 0.05, ***P* < 0.01, ****P* < 0.001, NS: *P* > 0.05).

**
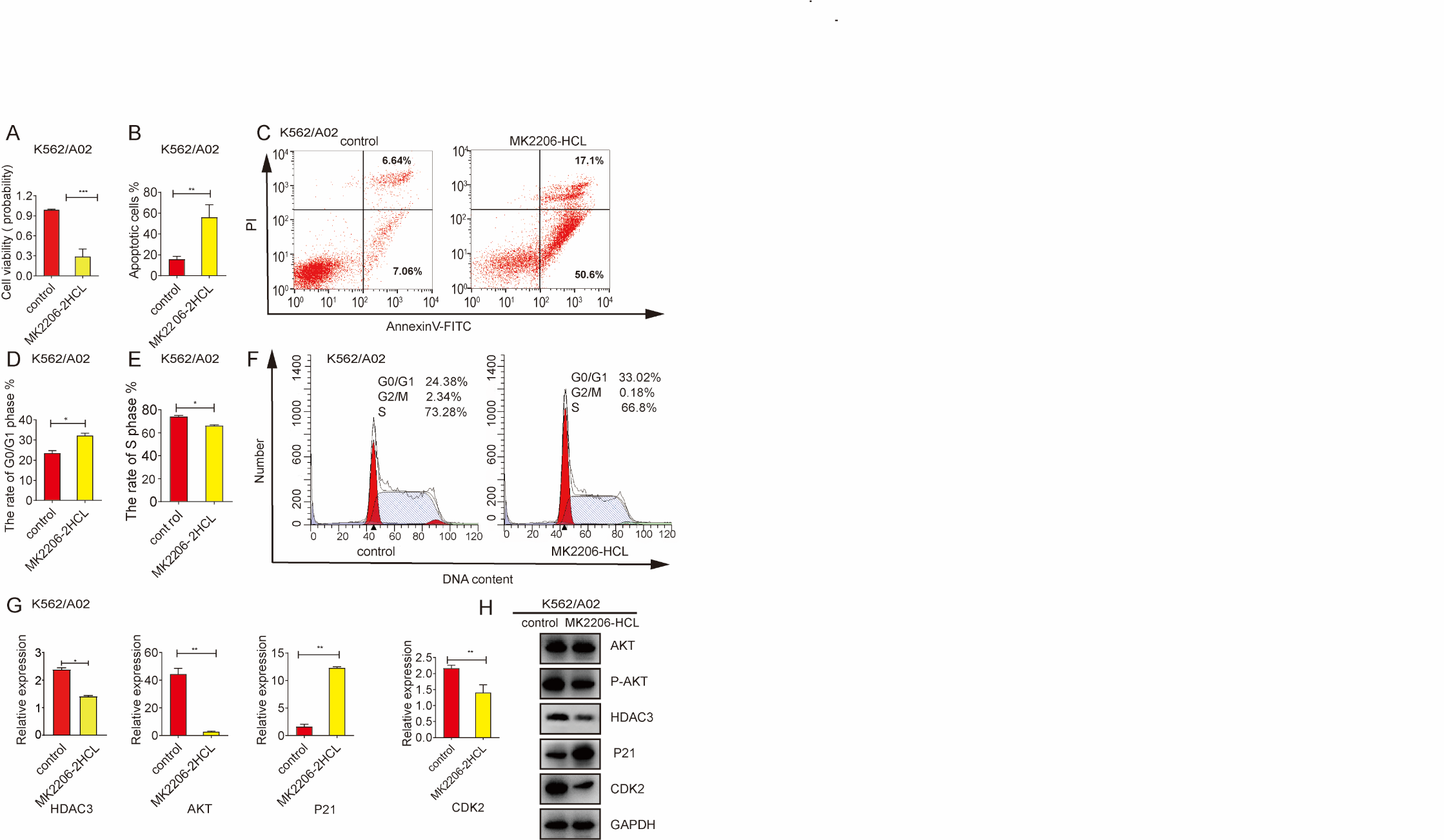
**

**Figure S7. AKT inhibitor reduces cell proliferation, increases cell apoptosis, induces cell cycle arrest at G0/G1 phase, and suppresses the expression of HDAC3, CDK2 and P21.** K562/A02 cells were incubated with an AKT inhibitor (MK2206-2HCL). **(A)** CCK-8 assays were used to assess cell proliferation ability. **(B)** Flow cytometry was used to analyze cell apoptosis. **(C)** is a representative of flow cytometry plot for detection of apoptosis. **(D-E)** Cell cycles of K562/A02 cells were analyzed by ﬂow cytometry after PI staining. **(F)** is a representative of flow cytometry for cell cycle detection. **(G)** Expression levels of HDAC3, AKT, P21 and CDK2 were measured by RT-PCR. **(H)** Expression levels of HDAC3, P-AKT, AKT, P21 and CDK2 were measured by Western blot. GAPDH was used as an internal control. Data represent three independent experiments, results are shown in the format, mean ± S.D. (**P* < 0.05, ***P* < 0.01, ****P* < 0.001, NS: *P* > 0.05).

**Supplementary Tables**

**Table S1.Comparison of different anthracycline-based chemotherapy regimens**

| Chemotherapy regimen | CR | PR | NR | OR | ORR | P |
| --- | --- | --- | --- | --- | --- | --- |
| Aclacinomycin-based regimens | 12 | 1 | 12 | 13 | 52% | ＞0.99 |
| Mitoxantrone-based regimen | 1 | 0 | 1 | 1 | 50% |  |

**Abbreviations:** CR, complete remission; PR, partial remission; NR, no response; OR, overall remission; ORR, overall remission rate.
